# Evolving Applications, Technological Challenges and Future Opportunities in Neuromodulation: Proceedings of the Fifth Annual Deep Brain Stimulation Think Tank

**DOI:** 10.3389/fnins.2017.00734

**Published:** 2018-01-24

**Authors:** Adolfo Ramirez-Zamora, James J. Giordano, Aysegul Gunduz, Peter Brown, Justin C. Sanchez, Kelly D. Foote, Leonardo Almeida, Philip A. Starr, Helen M. Bronte-Stewart, Wei Hu, Cameron McIntyre, Wayne Goodman, Doe Kumsa, Warren M. Grill, Harrison C. Walker, Matthew D. Johnson, Jerrold L. Vitek, David Greene, Daniel S. Rizzuto, Dong Song, Theodore W. Berger, Robert E. Hampson, Sam A. Deadwyler, Leigh R. Hochberg, Nicholas D. Schiff, Paul Stypulkowski, Greg Worrell, Vineet Tiruvadi, Helen S. Mayberg, Joohi Jimenez-Shahed, Pranav Nanda, Sameer A. Sheth, Robert E. Gross, Scott F. Lempka, Luming Li, Wissam Deeb, Michael S. Okun

**Affiliations:** ^1^Department of Neurology, Center for Movement Disorders and Neurorestoration, University of Florida, Gainesville, FL, United States; ^2^Department of Neurology, Pellegrino Center for Clinical Bioethics, Georgetown University Medical Center, Washington, DC, United States; ^3^J. Crayton Pruitt Family Department of Biomedical Engineering, Center for Movement Disorders and Neurorestoration, University of Florida, Gainesville, FL, United States; ^4^Nuffield Department of Clinical Neurosciences, University of Oxford, Oxford, United Kingdom; ^5^Biological Technologies Office, Defense Advanced Research Projects Agency, Arlington, VA, United States; ^6^Department of Neurosurgery, Center for Movement Disorders and Neurorestoration, University of Florida, Gainesville, FL, United States; ^7^Department of Neurological Surgery, Kavli Institute for Fundamental Neuroscience, University of California, San Francisco, San Francisco, CA, United States; ^8^Departments of Neurology and Neurological Sciences and Neurosurgery, Stanford University, Stanford, CA, United States; ^9^Department of Biomedical Engineering, Case Western Reserve University, Cleveland, OH, United States; ^10^Department of Psychiatry, Icahn School of Medicine at Mount Sinai, New York, NY, United States; ^11^Department of Neuroscience, Icahn School of Medicine at Mount Sinai, New York, NY, United States; ^12^Division of Biomedical Physics, Office of Science and Engineering Laboratories, Center for Devices and Radiological Health, United States Food and Drug Administration, White Oak Federal Research Center, Silver Spring, MD, United States; ^13^Department of Biomedical Engineering, Duke University, Durham, NC, United States; ^14^Division of Movement Disorders, Department of Neurology, University of Alabama at Birmingham, Birmingham, AL, United States; ^15^Department of Biomedical Engineering, University of Alabama at Birmingham, Birmingham, AL, United States; ^16^Department of Biomedical Engineering, University of Minnesota, Minneapolis, MN, United States; ^17^Department of Neurology, University of Minnesota, Minneapolis, MN, United States; ^18^NeuroPace, Inc., Mountain View, CA, United States; ^19^Department of Psychology, University of Pennsylvania, Philadelphia, PA, United States; ^20^Department of Biomedical Engineering, University of Southern California, Los Angeles, CA, United States; ^21^Physiology and Pharmacology, Wake Forest University School of Medicine, Wake Forest University, Winston-Salem, NC, United States; ^22^Department of Neurology, Center for Neurotechnology and Neurorecovery, Massachusetts General Hospital, Harvard Medical School, Harvard University, Boston, MA, United States; ^23^Center for Neurorestoration and Neurotechnology, Rehabilitation R and D Service, Veterans Affairs Medical Center, Providence, RI, United States; ^24^School of Engineering and Brown Institute for Brain Science, Brown University, Providence, RI, United States; ^25^Laboratory of Cognitive Neuromodulation, Feil Family Brain Mind Research Institute, Weill Cornell Medicine, New York, NY, United States; ^26^Medtronic Neuromodulation, Minneapolis, MN, United States; ^27^Department of Neurology, Mayo Clinic, Rochester, MN, United States; ^28^Department of Biomedical Engineering, Georgia Institute of Technology, Emory University School of Medicine, Emory University, Atlanta, GA, United States; ^29^Departments of Psychiatry, Neurology, and Radiology, Emory University School of Medicine, Emory University, Atlanta, GA, United States; ^30^Parkinson's Disease Center and Movement Disorders Clinic, Department of Neurology, Baylor College of Medicine, Houston, TX, United States; ^31^Department of Neurological Surgery, The Neurological Institute, Columbia University Herbert and Florence Irving Medical Center, Colombia University, New York, NY, United States; ^32^Department of Neurosurgery, Emory University, Atlanta, GA, United States; ^33^Department of Biomedical Engineering, University of Michigan, Ann Arbor, MI, United States; ^34^National Engineering Laboratory for Neuromodulation, School of Aerospace Engineering, Tsinghua University, Beijing, China; ^35^Precision Medicine and Healthcare Research Center, Tsinghua-Berkeley Shenzhen Institute, Tsinghua University, Beijing, China; ^36^Center of Epilepsy, Beijing Institute for Brain Disorders, Beijing, China

**Keywords:** deep brain stimulation, neuromodulation, epilepsy, Parkinson's disease, tremor, obsessive compulsive disorder, tourette syndrome, memory

## Abstract

The annual Deep Brain Stimulation (DBS) Think Tank provides a focal opportunity for a multidisciplinary ensemble of experts in the field of neuromodulation to discuss advancements and forthcoming opportunities and challenges in the field. The proceedings of the fifth Think Tank summarize progress in neuromodulation neurotechnology and techniques for the treatment of a range of neuropsychiatric conditions including Parkinson's disease, dystonia, essential tremor, Tourette syndrome, obsessive compulsive disorder, epilepsy and cognitive, and motor disorders. Each section of this overview of the meeting provides insight to the critical elements of discussion, current challenges, and identified future directions of scientific and technological development and application. The report addresses key issues in developing, and emphasizes major innovations that have occurred during the past year. Specifically, this year's meeting focused on technical developments in DBS, design considerations for DBS electrodes, improved sensors, neuronal signal processing, advancements in development and uses of responsive DBS (closed-loop systems), updates on National Institutes of Health and DARPA DBS programs of the BRAIN initiative, and neuroethical and policy issues arising in and from DBS research and applications in practice.

## Introduction

Neuromodulation, including cortical and subcortical approaches for management of neurological and neuropsychiatric disorders, continues to rapidly evolve. Technological advancements have enabled an increased understanding of neuronal signals involved in signs and symptoms of a number of neuropsychiatric conditions. The Fifth Annual Deep Brain Stimulation (DBS) Think Tank convened in Atlanta, GA, from May 19th to 21st, 2017 to address evolving applications, technological challenges and future opportunities in neuromodulation. This report highlights the challenges and opportunities addressed in the meeting. There was particular focus on technical developments, design considerations for DBS electrodes, emerging capabilities of responsive DBS (closed-loop systems), updates from the National Institutes of Health (NIH) and Defense Advanced Research Projects Agency (DARPA) DBS-based programs, focus upon advances and knowledge gaps in brain electrophysiology and sensor technology, and address of ongoing and newly arising neuroethical and policy issues generated in and by DBS research and uses in practice.

## Technical developments

### Technologies emerging due to investigator demand

Neuromodulation 2.0: Building a bridge from today's tonic pulse generators to tomorrow's adaptive neurological “co-processors.”

The burden of neurological disease has significant economic and societal impact. While neuromodulation-based therapeutic devices have forged significant in-roads in treating some neuropsychiatric conditions, overall, the relative success of such approaches has been limited by fundamental questions regarding the pathophysiology and mechanisms underlying both disease processes and DBS-based interventions.

**Figure 1:**
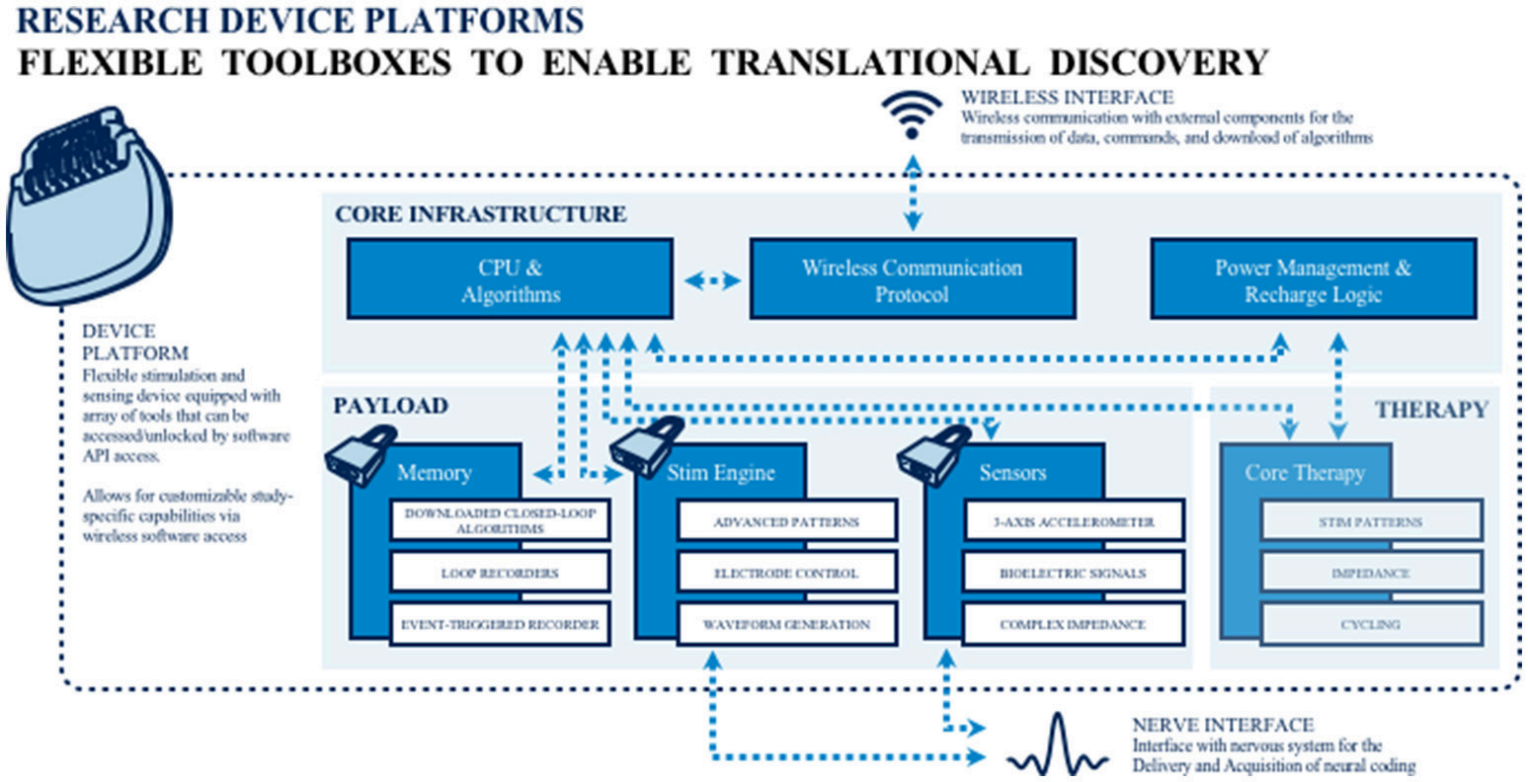
Toolboxes to promote translational neuromodulation research and discovery.

To address the extant knowledge gap, public and private teams are collaborating on building and deploying investigational research tools for studying the human nervous system in both health and disease. The primary goal of this work is to merge (basic and applied) biomedical research with engineering design methods to catalyze the development of next generation neuromodulation therapeutics (see Figure 1). The prototyping of therapeutic concepts from a dynamic systems perspective has fostered increased capability of bioelectronic systems that act more as a neurological “co-processor” to address neural system dysfunction, as opposed to the tonic, fixed-pattern stimulators used to date. Emergent technologies include devices that store event triggered recording and improved sensors capable of recording local field potential (LFP) from an increased number of channel combinations. Additional developments include an enhanced capacity to parse signals from the neural noise floor, development of embedded inertial sensors, use of advanced stimulation parameters and patterns, creation of a rechargeable battery (that can increase neurostimulator life to allow more long term applications when utilizing advanced research tools), production of upgradable and smaller devices, improved motion sensing and distance telemetry (e.g., real-time streaming) with higher throughput (not-compressed, multiple channels) and research development kits (greater customization, integration with local and distributed systems). External synchronization has been a challenge in closed loop devices, and implanted device technology with higher bandwidth than current systems has the potential to improve synchronization and to better detect the fast dynamics of neural signals of interest. Toward this end, newer networks can select recordings and set specific signals to improve synchronization. The potential release of second-generation devices (e.g., the Medtronic RC+S) for use in certain National Institutes of Health (NIH) projects funded under the United States' Brain Research through Advanced Innovative Neurotechnology (BRAIN) Initiative may address some of these needs. As well, the limited number of lead contacts for closed loop applications has been a constraint. Newer devices will likely facilitate the ability to sample two channels per lead; the use of additional channels could be beneficial to both increase sampling and to pair stimulation in closed-loop devices.

### Highlights and future directions

- Emerging technological developments include improved sensors, increased device memory, and rechargeable batteries.
- Development of external signals to improve closed loop synchronizations has been identified as being important to next-generation technology capability.
- An increased number of lead electrodes may improve both stimulation and recording capacities of both current and newly developing devices.

### Variable frequency stimulation as a new approach to DBS in defined applications

There are ongoing efforts to define particular signs and symptoms of neuropsychiatric disorders that may be mitigated by DBS intervention. Freezing of gait (FOG) is defined as a sudden arrest of forward stepping commonly affecting patients with advanced Parkinson's disease (PD) (Nutt et al., 2011). Occurrence and management of FOG is challenging as FOG is often resistant to levodopa treatment in patients with advanced PD. In small case series, low frequency stimulation (LFS) of the subthalamic nucleus (STN) (60–80 Hz) has provided short term benefit in reducing axial symptoms including FOG, although this has raised concerns about the worsening of appendicular symptoms, in particular tremor (Xie et al., 2015; Zibetti et al., 2016). A novel DBS paradigm that combined high and low frequency stimulation in varying patterns (VFS) to address FOG and appendicular PD motor symptoms has been proposed (Jia et al., 2017). Following an open label, doubled blind design, investigators utilized the PINS DBS system to assess the effect of VFS in FOG (NCT02601144). The study included twenty-eight (28) PD subjects presenting with FOG, who were implanted with bilateral STN DBS. At baseline, patients were evaluated under four DBS conditions: DBS off, LFS (60–80 Hz), HFS (130–180 Hz) and VFS for a total of 6 h. All subjects were discharged home on VFS settings and were scheduled for a follow-up second study visit after 6 months, and for a final evaluation at 12 months. Changes in the Unified Parkinson's Disease (UPDRS) motor scale and a 10-meter timed up-and-go (TUG) task were assessed at baseline, and following 1 h of high, low, and VFS. Chronic VFS therapy was assessed at 6 and 12 months with the FOG questionnaire and the gait and falls questionnaire. Initial data suggests that VFS was well tolerated and that it reduces appendicular symptoms and the number of FOG episodes compared to HFS and LFS at 12 months follow up. Finalized publication of the data is pending.

### Highlights and future directions

- A novel VFS DBS programming paradigm was shown to elicit improved benefit over traditional HFS and LFS programming in an open label design study.
- VFS was well tolerated and was shown to potentially reduce FOG episodes and improve TUG measures.
- The use of external sensing triggered by physiological changes indicative of FOG might be useful a specific biomarker to best utilize VFS.

### Biphasic DBS for essential tremor and dystonia

Despite advances in DBS techniques and technology, clinicians continue to face limitations in battery longevity and in stimulation-induced side effects. The conventional DBS waveform consists of a rectangular biphasic pulse, with an active, high-amplitude and short-duration phase, followed by a passive, low amplitude, charge-balancing phase. Pilot research at the University of Florida in movement disorders reported that square biphasic (sqBIP) pulses (with active rather than passive charge-balancing phase) were well tolerated and provided a greater clinical benefit when compared to commercially available DBS (Akbar et al., 2016).

An open label, pilot trial assessed (over a period of hours) the safety and tolerability of sqBiP DBS in patients with PD, ET, and dystonia (Almeida et al., 2017). Secondary analysis included effects on motor response produced by sqBiP DBS compared to conventional stimulation. Firmware was updated with proprietary software provided by the manufacturer (Medtronic). Patients were tested in at-home settings (conventional DBS), after a 30-min washout, and then at multiple time points after sqBiP DBS was implemented for a total of 3 h for ET and PD, and 2 h for dystonia.

At each evaluation, motor behavior and scales were videotaped, and accelerometer and GAITRite data were collected. There were no adverse events documented in either arm of the study. Significantly positive changes in tremor scores over time, and in accelerometer data were observed. Subsequent *post-hoc* analysis showed significance only between different time points and the washout period. For treatment of dystonia, there was a significant change in cervical dystonia scores, with *post-hoc* analysis revealing differences between other time points and the washout. Interestingly, there was a gradual improvement in GAITRite measures, including cadence, velocity, average step length, and double support time with sqBiP stimulation. sqBiP was well-tolerated in the acute ambulatory setting, with possibly similar benefits produced in motor scores and improved cadence, step stride, and double support gait assessments.

### Highlights and future directions

- A novel stimulation technique, sqBiP adjusted the pulse frequency and shape of pulses.
- In use against ET, sqBiP DBS improved tremor scales and accelerometer parameters. In treating dystonia sqBiP DBS produced an improvement in gait variables.
- Future studies will explore the mechanisms of sqBiP pulses; long term outcomes; battery consumption, and clinical benefits in treatment of ET, PD, dystonia and other select disorders.

### Distributed network control with high density neuromodulation technology for the treatment of intractable epilepsy

Anterior thalamic DBS (Salanova et al., 2015) and responsive neuromodulation (Bergey et al., 2015) for treatment epilepsy has been reported to show an approximately 65% reduction in seizures at long term follow-up. Preliminary data using an *in vitro* multi-electrode array in cell culture revealed that asynchronous multi-site stimulation eliminated synchronous epileptogenic activity. Furthermore, in animal models, multi-microelectrodes were more effective than macroelectrodes in terminating seizures through the use of asynchronous theta stimulation (Desai et al., 2016). While such animal studies are promising, it was suggested that the use of a non-human primate model of penicillin induced seizure) would be important (and thus is planned) prior to considering use of this approach in human patients.

A translational study using RC+S (Medtronic) is also currently planned to identify electrophysiological biomarkers and to integrate a closed-loop approach. The initial phase of our studies aims to use an external system to test stimulation in an open loop fashion using asynchronous distributed microelectrode theta stimulation. We will also record biomarkers and use these to design a closed loop neuromodulation algorithm. This will lead in phase 2 to a translational NHP study using RC+S (Medtronic) which will allow both open-loop and closed-loop algorithms based on the previous experiment. The third phase will translate the NHP findings into an early stage feasibility study in epilepsy patients. These studies rely on novel high-channel count electrodes, bidirectional neurostimulation devices and novel computational approaches.

### Highlights and future directions

- Preliminary data using *in vitro* multielectrode array in cell culture revealed asynchronous multi-site stimulation eliminated synchronous epileptiform activity.
- Multi-microelectrodes were more effective than macroelectrodes in terminating seizures in a rodent model using asynchronous theta stimulation.
- Using an acute non-human primate seizure model of epilepsy, the research will attempt to translate the above findings, and to identify biomarkers to be used in implementation of closed loop seizure control optimization.

## Advances in closed loop DBS

### Parkinson's disease

#### Closed loop DBS in PD

**Figure 2:**
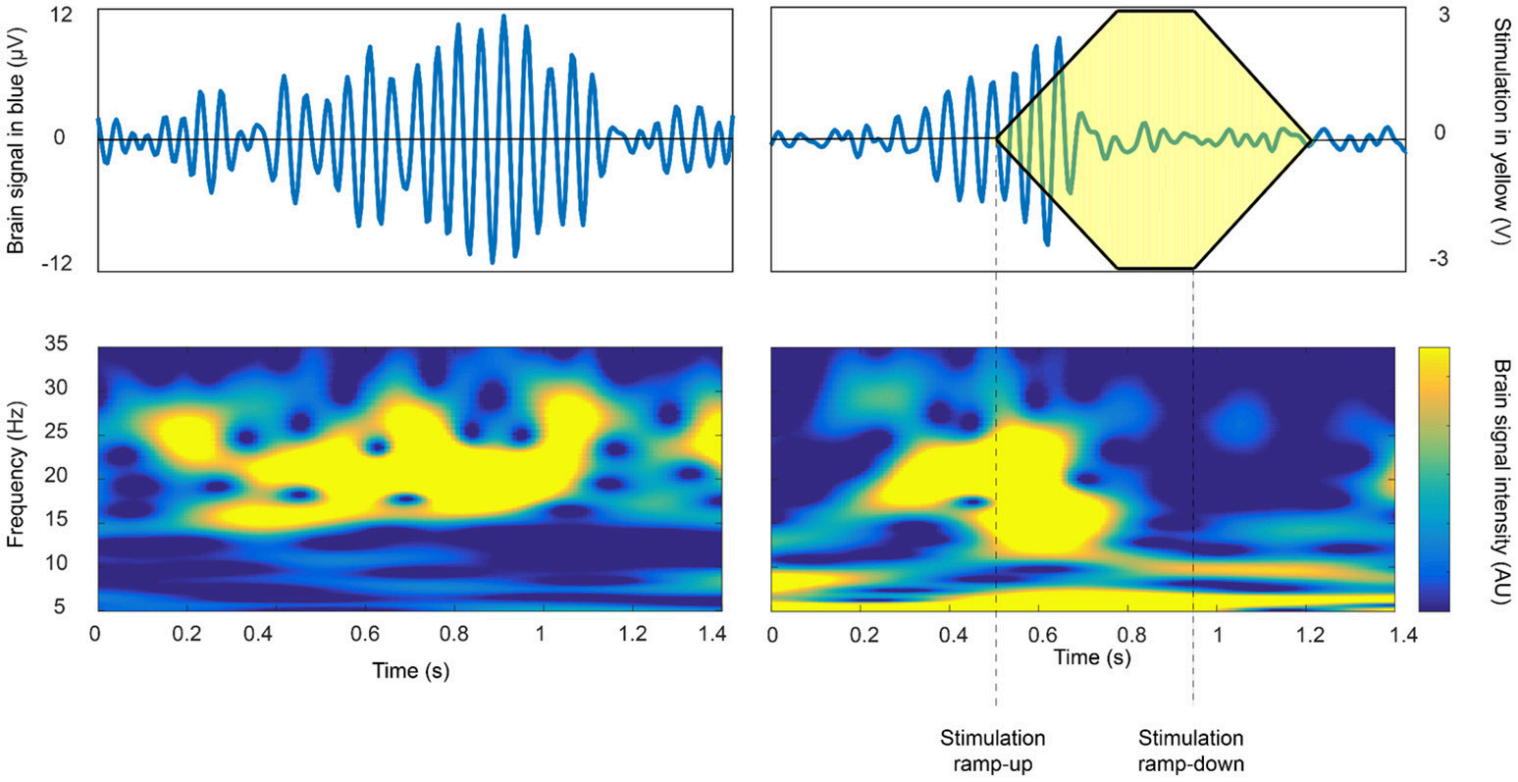
The beta burst duration and amplitude as a potential biomarker for closed loop approaches. On-off feedback control of beta activity recorded in the subthalamic nucleus in a patient with PD so as to terminate long duration, high amplitude beta bursts. Intermittent stimulation may be sufficient to improve motor impairment, whilst sparing more physiological periods of processing and thereby limiting side-effects (Tinkhauser et al., 2017 with permission).

Specific examples of the application of closed-loop DBS in PD are growing (Rosin et al., 2011; Little et al., 2013, 2016; Malekmohammadi et al., 2016). These cases have demonstrated symptom improvement, with substantial power savings and/or reduction in side-effects attributable to stimulation. The feedback substrates and closed-loop control algorithms involved have varied, but most have relied on the amplitude of beta activity as directly recorded in the basal ganglia-cortical loop. The amplitude of such beta activity correlates with bradykinesia and rigidity and is suppressed by both medications that exert effect on central dopaminergic activity, and high frequency DBS (Meidahl et al., 2017). Thus far, closed-loop control algorithms have either engaged an on-off activity pattern, with short ramping onset and offset (see Figure 2), or have employed a more gradual, proportional or hybrid control policy. One important consideration is the optimal reactivity of the closed-loop system, which may impact its efficacy, efficiency and ultimate therapeutic window. In healthy primates, and in patients with PD, beta activity is phasic (Tinkhauser et al., 2017). Longer bursts attain higher amplitudes, indicative of more pervasive oscillatory synchronization within the neural circuit. Shorter bursts predominate in healthy states; and in patients, the relative proportion of short and long bursts negatively and positively correlate with motor impairment, respectively. Therefore, it might be best to selectively terminate longer, (i.e., pathological) beta bursts through closed-loop DBS to both maximize power savings and to spare the ability of underlying neural circuits to engage in more physiological processing which may involve shorter bursts (Tinkhauser et al., 2017).

The role of more complex feedback signals, including multidimensional central and peripheral inputs, remains to be explored, as do the advantages of more sophisticated control algorithms. In particular, it may prove necessary to tailor control loops to afford improved amelioration of patient-specific patterns of impairment (Meidahl et al., 2017). On the other hand, one of the factors constraining the development of closed-loop DBS is the range of possible feedback signals, control policies, and stimulation patterns. Arguably, the field needs to focus on demonstrating an unequivocal gain over conventional DBS before closed-loop DBS techniques are further nuanced. The key will be to shift from acute trials in post-operative patients—where studies are confounded by stun effects and temporal constraints – to acute and then ultimately to chronic trials in patients who have already undergone conventional DBS. The latter will allow resolution of the stun effect, and will afford time for optimization of conventional DBS so that valid comparisons can be made. However, these types of chronic trials will require further development of enabling technology, together with a more informed understanding of the dynamics of target circuits.

#### Highlights and future directions

- In PD, beta activity is phasic with longer busts attaining higher amplitudes, indicative of more pervasive oscillatory synchronization within the neural circuit. Shorter bursts predominate in the healthy state, and the relative proportion of short and long bursts negatively and positively correlate with motor impairment, respectively.
- Termination of longer, pathological beta bursts through closed-loop DBS might maximize power savings, and spare the ability of underlying neural circuits to engage in more physiological processing.
- The next steps in adaptive DBS involves shifting from acute trials in post-operative patients to longer assessment and evaluation to minimize confounded factors.

#### Customizing control variables and control policy algorithms for closed loop DBS to treat tremor in Parkinson's disease

At present DBS is characteristically provided as continuous, open loop, non-responsive input, and uses a “one-size fits all” set of parameters for a wide range of neurological disorders. It cannot respond to the patient's state (asleep/awake, rest/active), dominant symptoms (tremor, bradykinesia, gait impairment) or medication level. In contrast, closed loop (i.e., responsive) DBS (CL– DBS) for PD, using kinematic and/or neural biomarkers, has the potential to deliver more precise and customized neuromodulation, based on state, symptom and level of medication. To be successful CL-DBS requires feedback signals (control variables) that are accurate reflections of the disease state or symptom, and neuromodulation paradigms (control policy algorithms) that are customized for that disease/symptom. Tremor is especially well-suited for responsive or CL-DBS as it varies in amplitude or presence over time and differs between PD subjects. CL-DBS for tremor can be approached using control variables such as a peripheral measure of tremor (Kinematic Cl-DBS) or the STN LFP beta band (13–30 Hz) power (Neural CL-DBS) (Malekmohammadi et al., 2016). Attenuation of STN beta band power by medication or DBS is associated with improvement in both rigidity and bradykinesia and has been the control variable used in CL-DBS studies to date (Kuhn et al., 2008; Whitmer et al., 2012). However, the presence of tremor itself may attenuate beta band power and thus Neural CL-DBS may not be useful for tremor (Shreve et al., 2017).

**Figure 3:**
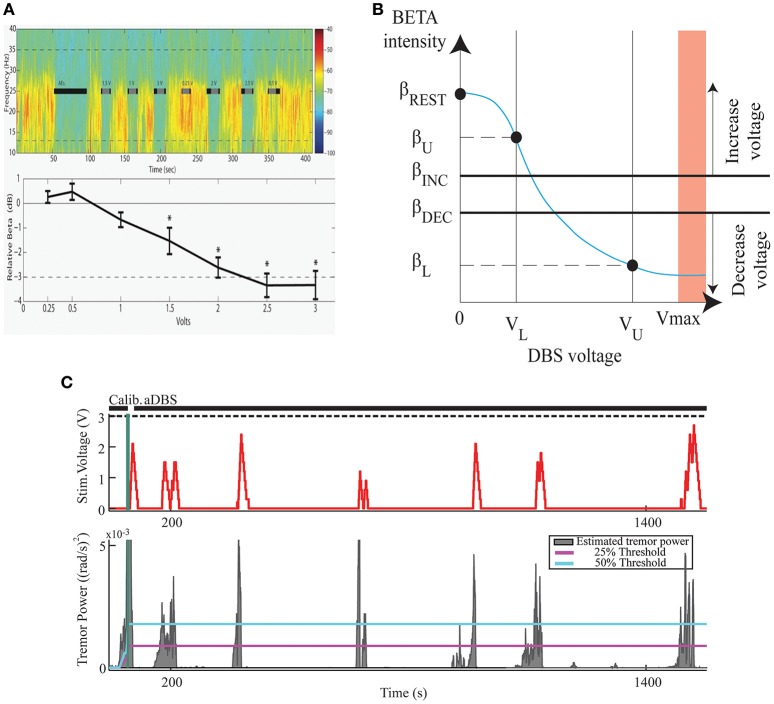
The Development of a Closed Loop System for PD Tremor. **(A)** Voltage dependent attenuation of STN beta band power; top panel- time frequency spectrogram, black bars indicate periods of STN DBS; lower panel group averaged relative beta band power at different DBS voltages. **(B)** Dual threshold control policy diagram; β_REST_–beta power no DBS at rest; β_U_, β_L_–beta power at lower and upper limits of DBS voltage; β_INC_, β_DEC_–beta power of upper and lower thresholds. **(C)**. Example of STN KCL-DBS using dual tremor power thresholds (lower panel blue and magenta lines). DBS voltage (upper panel) follows tremor power and remains off for much of the trial when there is no tremor.

The effectiveness and efficiency of Kinematic compared to Neural CL-DBS and to open loop DBS were determined in six PD subjects (Malekmohammadi et al., 2016) using a dual threshold control policy algorithm, (Figure 3), for either beta band power, (Figures 3A,B), or tremor power, measured by a Bluetooth enabled smartwatch (Figure 3C). Voltage was increased if either beta or tremor power was above the upper threshold, remained the same if between thresholds, and was decreased if below the lower threshold. Both techniques improved tremor to a similar degree; Kinematic CL-DBS used 10.6% of the total energy that would have been delivered (TEED) during open loop DBS and Neural CL-DBS used 31.5% TEED. Kinematic CL-DBS was significantly more efficient than Neural CL-DBS (*P* < 0.05). Neural CL-DBS was efficacious in treating tremor if the initial voltage was in a therapeutic range; whereas Kinematic CL-DBS required tremor to be present and initial voltage was set at the lower therapeutic limit. This research demonstrates that CL-DBS, using either a neural or kinematic control variable, is more efficient than open loop DBS. Furthermore, it highlights the potential that additional development of patient- and symptom-specific control variables, and control policy algorithms will improve the efficiency, efficacy and specificity of DBS therapy for PD.

#### Highlights and future directions

- Tremor in PD is a symptom especially well-suited for responsive or closed loop DBS as it varies in amplitude or presence over time and differs between patients.
- In a pilot trial, there were no differences between tremor control with adaptive DBS using Kinematic or Neural CL-DBS and Kinematic CL-DBS was significantly more efficient than Neural CL-DBS KCL-DBS.

#### Closed-loop DBS for freezing of gait in PD

Levodopa-resistant posture, and FOG symptoms are disabling and difficult to address in patients with PD. Recent DBS trials have rarely addressed FOG and specifically “on-medication” freezing and falling. Freezing appears to involve some GABA-ergically-mediated activity of the globus pallidus interna (GPi) that leads to dysfunction of the pedunculopontine nucleus (PPN). Human DBS studies have targeted the PPN or the PPN plus STN with mixed—and in many cases, unsatisfactory outcomes (Stefani et al., 2007; Moreau et al., 2009; Moro et al., 2010). However, there is no consensus regarding the best PPN target and both rostral and caudal pedunculopontine nucleus subregions have been targeted (Thevathasan et al., 2017). There is great variability in clinical methodology used among surgical centers and the spread of stimulation and inconsistency in targeting suggests that neighboring brain stem regions may be implicated in any DBS response. Because of the intermittent nature of FOG, a feasibility closed-loop neuromodulation (CL-DBS) approach for bilateral PPN DBS plus conventional bilateral open-loop DBS (OL-DBS) of the GPi to manage on-medication FOG in PD has been initiated at the University of Florida. Five patients with advanced PD and refractory FOG were implanted with Medtronic Activa PC+S implantable neurostimulators leads. The patients were carefully evaluated, so as to define true “on” medication freezers. A closed-loop PPN DBS paradigm has been developed using LFPs occurring in GPi and PPN during normal walking and during maneuvers known to trigger freezing episodes. Assessments are blinded and videotaped including objective gait laboratory analysis. PPN CL-DBS was aimed to deliver stimulation at different frequencies (5, 25, 65, and 130 HZ) at the onset of gait. This PPN CL-DBS paradigm was also used for long-term PPN CL-DBS via the Nexus-E firmware, which allows similar Nexus-D operation, but is a completely embedded, enclosed system with no external triggers or machines. GPi stimulation settings were determined by clinical benefit consistent with current standard of care for optimizing pallidal DBS. The study remains ongoing and will report safety and feasibility of this approach along with clear neurophysiological changes in beta and theta bands that could be potential biomarkers to guide future adaptive DBS approaches.

#### Highlights and future directions

- A novel closed loop DBS system that targets GPi and PPN is currently investigated for treatment of refractory “on” FOG in PD patients. Approach appears safe and applicable.
- Challenges will include establishing the most effective location of PPN contacts, and programming settings, identifying reliable neurophysiological biomarkers of walking and FOG, and refining patient selection in light of wide variability among advanced PD patients.

#### Closed loop deep brain stimulation for PD: update on use of cortical control signals

Currently used DBS devices generally deliver “open loop” stimulation, continuously stimulating target structures regardless of changes in the brain circuits related to disease expression. Device programming is a labor-intensive process that is based on “trial and error” and requires significant clinical expertise, which are barriers to widespread clinical application. Additionally, continuous open-loop DBS for PD may result in suboptimal control of fluctuating motor signs, stimulation-induced adverse effects, and short battery life (Little et al., 2016).

**Figure 4:**
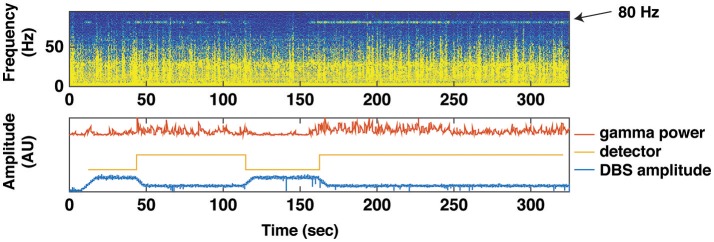
Using Gamma Power as a Biomarker for Closed Loop Deep Brain Stimulation. A brief trial of closed loop stimulation using a cortical detector in a patient with intermittent dyskinesia in spite of therapeutic STN DBS. This trial was implemented with data streaming from PC+S to an external computer (using Nexus D2/3). Top: spectrogram of motor cortex ECoG signal over 300 s, showing several epochs of an 80 Hz oscillation heralding the dyskinetic state. Bottom: transitions in stimulation triggered by the 80 Hz oscillation. Red line depicts the gamma power envelope at 80 Hz center frequency and 5 Hz bandwidth. Gold line depicts the detector that classifies gamma power to high or low values. Blue line depicts spectral power at stimulation frequency (160 Hz). When average gamma power (over 10 s) exceeded or dropped below a threshold (2.5 × standard deviation of gamma power during a calibration recording), stimulation was decreased, or increased, respectively.

As previously noted, one approach is to utilize subthalamic beta band activity as the control signal for closed loop stimulation (Little et al., 2013). Advantages of this approach include conceptual simplicity, and that the control signal can be derived from the same electrode array as used for therapeutic stimulation, thereby obviating the need for additional sensor electrodes. Disadvantages include a relatively low amplitude signal with high stimulation artifact, and that the beta band activity is strongly affected by normal movement, not just the severity of Parkinsonian motor signs (Qasim et al., 2016). We have developed an alternative technique for closed loop stimulation in PD using a sensor that is permanently implanted in the subdural space over the primary motor cortex. This is attached, along with the ipsilateral subthalamic stimulating lead, to an investigational pulse generator (Activa PC+S, Medtronic) with sensing as well as stimulation capability that can be used to prototype feedback control algorithms. The control strategy is based on detection of a narrow band gamma oscillation (60–90 Hz) that has previously been shown to be a biomarker of the dyskinetic state (Swann et al., 2016) (Figure 4).

We have tested this method in three phases (1) *in vitro* experiments using previously collected cortical signals converted to analog voltages and applied to a saline bath containing a paddle lead attached to an external PC+S; (2) in the clinic, streaming data from an implanted PC+S to an external laptop that controlled the PC+S stimulus via radiotelemetry (Herron and Chizeck, 2014), and (3) in a second clinical application, which evaluated the control algorithm totally embedded in PC+S. In two test subjects, closed loop control (Nexus E) provided 26 and 39% reduction in energy delivered, without adverse effects. Patients in this study did not perceive stimulation- related changes. Cortical contacts were implanted through the same DBS burr hole and appeared to be stable in the long-term (as observed in other recent studies conducted at multiple sites).

#### Highlights and future directions

- A new technique aims to optimize electrophysiological signals used for closed loop DBS using a sensor permanently implanted in the subdural space over primary motor cortex.
- Utilizing an investigational pulse generator (Activa PC+S, Medtronic), the control strategy is based on detection of a narrow band gamma oscillation (60–90 Hz) previously shown to be a biomarker of the dyskinetic state.
- Future challenges include identifying the most effective type of detection; selecting the best type of signal; noise reduction; identifying the ideal frequency band; and discerning between use of single vs. multiple frequencies.

### Deep brain stimulation for other neuropsychiatric conditions

#### Tourette syndrome

Tourette syndrome (TS) is a developmental neuropsychiatric disorder characterized by involuntary and vocal tics, which is commonly co-morbid to other conditions, such as attention deficit hyperactivity disorder and obsessive compulsive disorder (Cheung et al., 2007; Kenney et al., 2008). The etiology of TS remains largely unknown; however, a commonly accepted hypothesis implicates dysfunction of corticostriatal and thalamocortical circuits (Albin and Mink, 2006). DBS is an emerging therapy for cases of severe and intractable TS, and next generation DBS devices, such as the Neuropace RNS and Medtronic Activa PC+S, provide tools with which to record electrophysiological signals that can be used to study network effects and pathophysiology in this disorder.

Two studies of TS electrophysiology using DBS have recently been conducted at the University of Florida. The first implanted dual DBS leads in the centromedian- parafascicular complex of the thalamus, and employed a NeuroPace RNS device both to study electrophysiology of tics and to develop a responsive neurostimulation paradigm. Responsive DBS was achieved in one subject, delivering stimulation in response to increases in low-frequency activity (5–15 Hz) in the thalamus that correlated with dystonic tics (Molina et al., 2017). A second study, using the Medtronic Activa PC+S devices targeted the same thalamic nucleus bilaterally, but also included bilateral cortical subdural strips over the motor cortex to study the thalamocortical network of tic generation (Shute et al., 2016).

Long complex tics were shown to be concurrent with a consistently detectable low frequency activity in the thalamus, as shown in other studies. However, stimulation artifacts resulted in the development of a responsive DBS based on the cortical signatures of tic generation, which involved increases in cortical beta activity (Shute et al., 2016). An ongoing NIH study is examining the physiology of tics and closed loop DBS in 10 human subjects. Resetting tic mechanisms to achieve a more normal pattern of oscillatory activity may reduce tics and also lead to less device discharge over longitudinal follow-up.

#### Highlights and future directions

- Pilot studies of responsive neurostimulation in TS patients have been conducted and a larger trial is currently underway.
- Neural activity precedes tic onset, and successful stimulation results in cortical phase amplitude coupling.
- Future challenges—and opportunities—include developing consistent programming algorithms, improving stimulation settings, achieving higher resolution tic detection, and defining additional biomarkers.

#### Optimizing neurophysiologic DBS signal for treatment of tourette syndrome

LFP analysis is a well-established method for investigating disease, and network and stimulation dynamics in PD. Analysis of intraoperative basal ganglia LFPs can potentially predict the optimal stimulation contact (Ince et al., 2010). There is increasing interest in the use of DBS to treat signs and symptoms of TS, and multiple DBS targets have been proposed for the treatment of TS (Viswanathan et al., 2012). LFP analysis using signals captured from a combination of DBS electrodes and cortical recordings has previously shown thalamocortical activity changes during tics in TS (Shute et al., 2016). Limited data exist about the role of LFPs in the posteroventral GPi in TS, although open label stimulation at this site does seem to improve symptoms (Shahed et al., 2007).

LFP recordings from the GPi during an intraoperative testing paradigm capturing the resting state, voluntary movements, and tic activity have been analyzed and reported in 3 subjects with TS (Jimenez-Shahed et al., 2016). The LFP data filtered between 13 and 30 Hz indicated the presence of event related desynchronization with lower amplitude beta band oscillations during tic periods compared to the resting state. During tic periods, there was also amplitude enhancement in the gamma range (40–150 Hz) and higher frequencies (150–500 Hz) in all subjects. Subjects demonstrated individual changes in the spectral power of LFPs in both the low and high frequency oscillations (HFO) during different states. The resting state was further characterized by coupling between the phase of theta-low alpha oscillations to the amplitude of HFOs in two subjects, while tic activity was associated with beta-HFO phase-amplitude coupling (PAC) in all 3 subjects. These results were in contrast to the novel identification of beta-HFO PAC in the GPi of four un-medicated PD patients at rest. These findings suggest that tic activity can be neurophysiologically distinguished (at the subcortical level) from rest, voluntary movements and akinesia, and that the GPi is a viable target for neuromodulation to decrease tics in TS. Additionally, these data challenge the view that beta-HFO PAC is only a marker of akinesia, since tics represent a hyperkinetic state. These results also highlight the importance of investigating the HFO range of LFP activity.

Given these findings, we posit that continuing investigation of the LFP spectral characteristics and non-linear interactions between different LFP frequency bands across nodes within the basal ganglia-thalamo-cortical network in TS (and other movement disorders) will broaden understanding of the neurophysiologic abnormalities characterizing these conditions. This knowledge will also contribute to the identification of the most appropriate and sensitive signals to trigger closed loop stimulation when different movement patterns are present.

#### Highlights and future directions

- LFP recordings from the GPi demonstrate individual changes in the spectral power in both the low and HFO sub-band during different rest, voluntary movements or tics.
- Tic activity was associated with beta-HFO phase-amplitude coupling in this study.
- Continued investigation of the LFP spectral characteristics of different movement disorders will contribute to the identification of the most appropriate and sensitive signals to trigger and to develop closed loop stimulation.

#### Studying oscillatory activity in deep brain stimulation for depression

DBS of the subcallosal cingulate white matter (SCCwm) has been shown to elicit durable improvements in depressive symptoms (Riva-Posse et al., 2017). Here, preliminary work is presented that characterizes network-level electrophysiological changes in patients with treatment-resistant depression who were implanted with SCCwm-DBS. Using a prototype bi-directional DBS platform that employed the Activa PC+S (Stanslaski et al., 2012) in conjunction with dense array EEG, preliminary evidence of electrophysiologic changes in the SCC and downstream cortical regions was demonstrated that correlated with both the disease state and stimulation conditions.

In order to assess the validity of any oscillatory biometric captured from patients with chronically implanted prototype devices, preliminary evaluations focused upon limitations of oscillatory analyses in the context of technical device capabilities. In particular, it is known that amplifier limitations are sensitive to disease-independent tissue impedances and impedance changes. Thus, amplifier-related distortions present in bi-directional DBS devices during stimulation were modeled in order to identify potential spurious oscillatory results. Non-linear gain responses from an amplifier that is engaged to its output limits can result in strong high-frequency oscillations and low-to-high phase-amplitude coupling spuriously because of soft-clipping of the input signal. In light of this, we are developing an interactive tool that can be used to assess level of gain compression making analysis and interpretation of oscillatory results more reliable.

Modern analytical approaches were employed to overcome challenges that result from both recording noise and low patient sample sizes. Next, we utilized a machine learning-based algorithm to extract oscillatory biometrics in the chronic PC+S recordings. This approach, when learned on limited, noisy data in a training set, is able to predict the current level of depression severity (as measured by weekly rating scales) with approximately 50% correlation in a testing set of patients. This strategy appears to have technical, analytical, and practical advantages given the limited number of patients, sparseness of sampling, and the known trajectory of clinical depression response(s). This approach, when complemented with standard Fourier-domain approaches, can help to identify useful models for biometrics construction that can be based on correlative analyses.

Despite promising initial results, the exact mechanism of action of SCCwm-DBS still remains unclear, but appears to involve cortical regions beyond the site of stimulation (Mayberg et al., 2005). Using multimodal electrophysiology, we are therefore investigating rapid, transiently induced cortical oscillatory responses to connectomics-optimized SCCwm-DBS. In all patients with simultaneous PC+S LFP and dense EEG, alpha power change patterns can be used to clearly separate stimulation at the optimal SCC contact from stimulation at an adjacent contact on the DBS lead just 1.5 mm away. The complementary accuracy and reliability of this method in confirming current tractographic approaches is the focus of ongoing studies employing a larger cohort.

#### Highlights and future directions

- Various approaches can be used to identify putative biometrics of depression and anatomic sites and networks that be employed in future closed-loop DBS to objectively confirm proper anatomical targeting and potentially optimize therapeutically effective parameters of DBS in treatment of depression.
- Assessment, iterative modification, and expanded testing-set validation will be continued in ongoing investigations with additional subjects.
- Future opportunities will focus upon identifying stimulation settings that will reproduce the putative biomarkers observed on EEG.

#### Central thalamic stimulation for traumatic brain injury (TBI)

Involvement of the central thalamus has been implicated in the pathophysiology of certain types of traumatic brain injury (TBI). As this area links to other relevant brain networks and regions, the involvement of the central thalamus may play an important—and reciprocal—role in other brain responses to TBI, as well. Neural networks mediating arousal project to the central thalamus, with efferent projections to the anterior forebrain (Schiff, 2016). Pre-clinical animal (non-human primate—NHP) and rodent) models have elucidated these central thalamic to forebrain connections and their functional activation using electrophysiologic methods and optogenetic techniques combined with fMRI (Liu et al., 2015; Baker et al., 2016). Proof of concept that DBS of the central thalamus can produce improvements in cognitive performance and arousal supports the further development of clinical studies (Giacino et al., 2012). Additional studies in human patients with minimally conscious state showed that central thalamic DBS produced improvement(s) in both motor and cognitive function. Notably, a single-subject in minimally conscious state demonstrated motor and cognitive improvement with central thalamic DBS; this patient regained capacity to identify objects and speak after an initial titration phase along with improvements in attention and organized activity of the upper limb. Oral feeding was greatly improved with DBS as well. A clinical trial targeting patients with persistent cognitive difficulties after TBI is planned. Six patients with severe to moderate brain injury (GOSE Outcome level 6–7) at least two years following their initial injury with persistent cognitive impairment limiting regaining vocation and social reentry will be enrolled. The primary outcomes measure will test attention and working memory function. EEG will be used as a secondary physiological marker of cognitive impairment in order to examine physiological mechanisms of DBS effects and to potentially develop adaptive technologies.

#### Highlights and future directions

- Preclinical and clinical data support the role of the central thalamus and its projections in arousal and attention.
- A clinical trial aiming to assess the effect of central thalamic DBS in patients with TBI is underway. Cortical recordings and correlates of cognitive function will be explored using recording DBS technology and EEG.
- Challenges include establishing definitive anatomical substrate(s) for stimulation, selecting ideal candidate patients, obtaining neuroimaging assessments, and developing and implementing the most effective and reliable programming techniques.

#### Adaptive DBS for obsessive compulsive disorder (OCD)

Ventral Striatum (VS) DBS has FDA humanitarian device exemption approval for treatment of intractable OCD, and has been shown to incur 60% clinical benefit in recent meta-analysis (of open-label studies) (Alonso et al., 2015). However, management and programming strategies are increasingly challenged to provide persistent benefit while limiting the occurrence of DBS-induced behavioral side effects, most notably hypomania (Widge et al., 2016). Programming adjustments have been made largely on the basis of acquiring acute beneficial effects on outcome measures of “anxiety reduction,” “improved mood” and “increased energy.” Induction of “mirth” has served as a guidepost measure to programming, and has been regarded as predictive of good response to treatment (Haq et al., 2011). However, DBS-induced mirth also represents a potential risk for development of hypomania or mania. Thus, there is a clear need for an adaptive DBS (aDBS) system that can correctly assess hypomania as distinguished from a euthymic mood state, and automatically adjust stimulation accordingly.

A pilot study funded by the NIH BRAIN Initiative aims to develop and to test a prototype aDBS system for intractable OCD that uses LFP signals to automatically adjust DBS parameters to both improve symptom management and reduce stimulation-induced behavioral side effects. One of the challenges of this study was to identify a label for the classifiers that was objective, reliable, and fitting the National Institute of Mental Health (NIMH) Research Domain Criteria (RDoC) Constructs. Positive Valence (e.g., for elevated or euthymic mood) and Negative Valence Constructs (e.g., to encompass depression, anxiety, and disgust associated with worsening OCD) were selected, with facial affect being used to represent the motor output of emotional state; a measure considered to be superior to clinician ratings of affect for tracking changes in real time. The automated facial affect recognition (AFAR) platform (Tian et al., 2001) will be utilized, and time-locked with inputs from LFPs, EEG, motion, physiology, and changes in DBS programming in order to build classifiers for use in this, and future studies.

#### Highlights and future directions

- Ongoing studies are underway toward the development of adaptive technology that can provide accurate classification of acute fluctuations in obsessive ideation and/or compulsive behaviors that can be used to improve DBS programming for use in treatment of OCD.
- The use of an automated facial recognition program will be evaluated as a potential biomarker of emotional state and to determine feasibility for use in adaptive control.
- Challenges include determining how the system can separate physiologic from pathological states, and if and how the system can adequately classify numerous behavioral signals.

#### Brain state monitoring for closed loop epilepsy

Epilepsy is an epidemiologically common neurological disease and over 1/3 of patients have drug resistant epilepsy. In select cases, neurosurgical intervention can cure focal epilepsy, but many patients are not surgical candidates because their seizures are poorly localized or originate from brain regions that cannot be safely resected. Seizures are generally treated as random events, and there are currently no proven surrogate biomarkers for seizures. Clinicians must select a treatment and titration plan (drug, dose, stimulation parameters, etc.) based on clinical guidelines, and then wait for a treatment failure to make further treatment adjustments.

Advances in neural engineering have led to implantable devices capable of therapeutic electrical stimulation (Fisher and Velasco, 2014), seizure detection and forecasting (Cook et al., 2013). Electrical stimulation of the anterior nucleus of the thalamus (Fisher et al., 2010; Salanova et al., 2015) and responsive stimulation of detected electrophysiological abnormalities (Morrell and RNS System in Epilepsy Study Group, 2011; Heck et al., 2014; Bergey et al., 2015) were shown to reduce seizure burden in well-designed pivotal trials, but patients rarely achieved complete seizure freedom. Recently, multiple groups have demonstrated the feasibility of seizure forecasting in both humans (Park et al., 2011; Cook et al., 2013; Brinkmann et al., 2016) and canines (Howbert et al., 2014; Brinkmann et al., 2015) using intracranial EEG. Seizure forecasting has also been demonstrated in ambulatory patients with an implantable device that provides continuous intracranial EEG (iEEG) integrated with a personal assistant device (PAD), running both seizure detection and forecasting algorithms (Cook et al., 2013). Next generation implantable devices are now poised to transform epilepsy management by integrating brain sensing, active brain probing, seizure detection, electronic seizure diaries, seizure forecasting, and intelligent therapeutic stimulation.

The fact that seizure probability fluctuates opens the possibility of directly tracking seizure probability and dynamically adjusting therapy to prevent seizures. Furthermore, it has been shown that the neuronal assemblies activated during seizures may be consolidated in post-seizure slow-wave sleep, i.e., physiological learning mechanisms may strengthen the seizure engram (Bower et al., 2015). This observation suggests targeting neuronal dynamics during post-seizure slow-wave sleep as a brain state dependent therapy. Thus, devices providing continuous brain sensing, probing, and intelligent stimulation based on seizure probability and brain state can open new therapeutic options. In addition, the ability to directly interact with the nervous system could open a new era of brain research.

**Figure 5:**
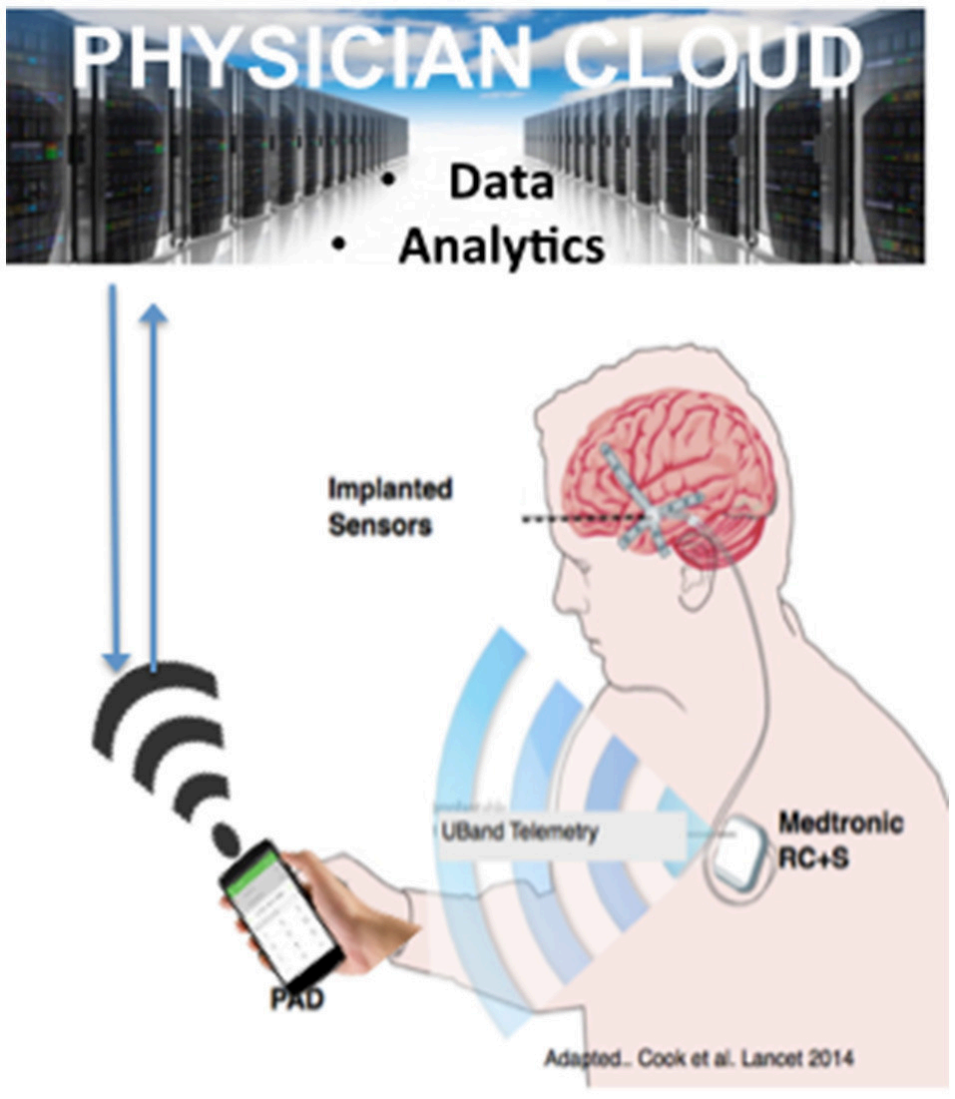
Next Generation Epilepsy Therapy Platform. The implanted, rechargeable Medtronic RC+S provides bi-directional coupling with the brain in patients with epilepsy. The personal assistant device (PAD) provides off-the-body analytics and is bi-directional with a cloud environment. The figure is adapted from Cook et al. with permission (Cook et al., 2013 with permission).

For example, the RC+S (Bourget et al., 2015) is a rechargeable device, with sensing, stimulation, embedded computational payloads, and continuous iEEG telemetry that affords capability of distributed computing & analytics on a hand-held PAD and cloud environment. It is also rechargeable. Currently pre-clinical development and validation of RC+S in dogs with naturally occurring epilepsy is underway and a human feasibility trial is planned for early 2019 (Figure 5).

#### Highlights and future directions

- There is a need for developing better tools to precisely identify and analyse seizures. Neuronal ensembles during seizures using scalp EEG have shown that neurons that fire together (at the onset of seizure), wire together (during slow wave sleep).
- Utilizing advanced DBS devices, a currently initiated preclinical study aims to continuously stream EEG data to detect anomalies in epilepsy, and improve seizure forecasting and detection.

## DBS electrodes

### Electrochemistry of deep brain stimulation electrodes

**Figure 6:**
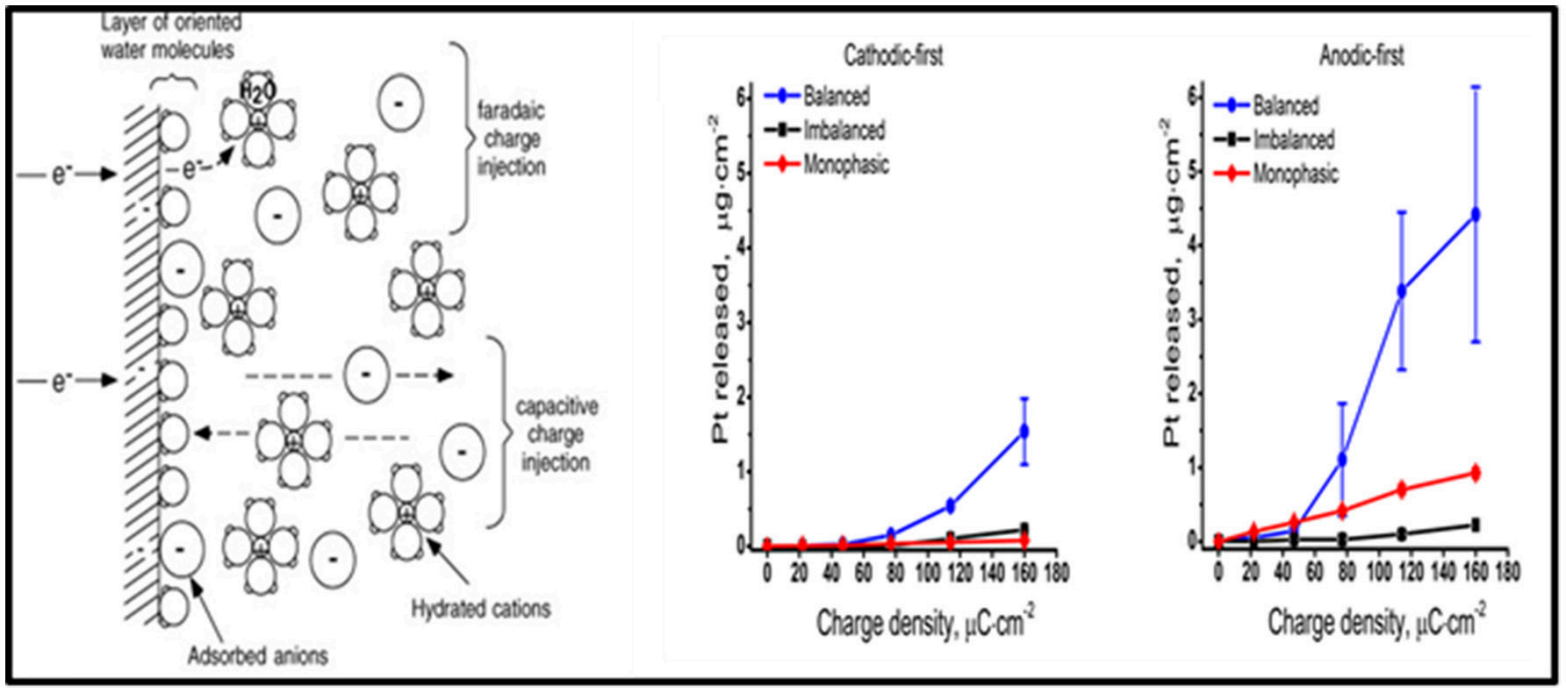
The electrode-electrolyte interface. A schematic representation of the electrode-electrolyte interface [from (Merrill et al., 2005 with permission) far left]. The concentration of Pt measured at different charge densities for cathodic-first (middle) and anodic-first (far right) stimulation waveforms (Kumsa et al., 2016 with permission).

The purpose of DBS electrodes is to artificially manipulate neural activity by generating an electric field in the tissue, ultimately resulting in a redistribution of charged particles in the extracellular space. Ideally, the electrode charge is injected capacitively, so that electrons are not transferred between the electrode-tissue interface and charge is rearranged in the tissue in response to the injected charge on the electrode side. The amount of charge injected is transferred via an electron transfer process (i.e., a faradaic reaction), and capacitive reactions occur as well (Merrill et al., 2005). Undesired faradaic reactions include electrode dissolution products that diffuse into tissue (Figure 6).

Balancing the amount of charge injected during the stimulation phase by a subsequent phase of the opposite polarity was formerly thought to avoid undesirable faradaic reactions. However, imbalanced charge biphasic waveforms are now known to reduce the amount of platinum (Pt) electrode dissolution when compared to balanced charge biphasic waveforms (Kumsa et al., 2016). In addition to minimization of Pt dissolution, imbalanced charge biphasic waveforms extend the parameter space that could be explored for current steering to selectively activate a target brain region during DBS therapy. More often than not, selection of stimulation parameters is made under consideration for thresholds of tissue damage. While there is no recognized standard addressing safe levels of stimulation, the Shannon plot has been used to set charge and charge density limits. Acute animal data collected used to establish the Shannon plot implies that tissue damage might be dependent on charge injection and surface area of the electrode used. These considerations may guide future selections of electrode materials and/or stimulation parameters.

### Highlights and future directions

- Design considerations for DBS electrodes should incorporate the needs of different spatial and temporal resolutions.
- Charge considerations include safety limits for charge density to avoid tissue damage that can be further characterized by animal studies to demonstrate:
  ◦ Shannon plots of tissue damage thresholds (30 uC/cm^2^ warning derived from Shannon plot at 50 Hz); and
  ◦ Charge-balanced biphasic waveforms.
- The threat of stimulation-induced damage has been shown to be dependent on charge injection and the surface area of the electrode.

### Biophysics of recording through deep brain stimulation electrodes

LFP recordings from DBS electrodes represent an exciting opportunity to study pathological activity in neurological disorders and to provide a potential control signal for closed-loop DBS systems. In PD, these LFP recordings have shown pathological hyper-synchrony within the beta frequency range (e.g., 13–30 Hz) and the oscillations can be reduced with therapeutic DBS (Kuhn et al., 2008; Bronte-Stewart et al., 2009). Closed-loop stimulation using the beta-band LFP as a control signal has demonstrated the potential to improve both the efficiency and possibly the efficacy of DBS (Little et al., 2013; Rosa et al., 2015). New implantable systems can also perform both stimulation and recording through DBS leads (Kuhn and Volkmann, 2017).

Although LFP recordings have the potential to provide disease biomarkers and a control signal for closed-loop stimulation, the origin of the LFP is poorly understood. Because clinical applications of the LFP (e.g., to optimize implant location) exploit the spatial dimensions of the LFP and relative changes in its frequency content (Zaidel et al., 2010), successful application of LFP recordings requires accurate interpretation of the source, recording volume, and various experimental factors (e.g., non-ideal properties of the recording system).

**Figure 7:**
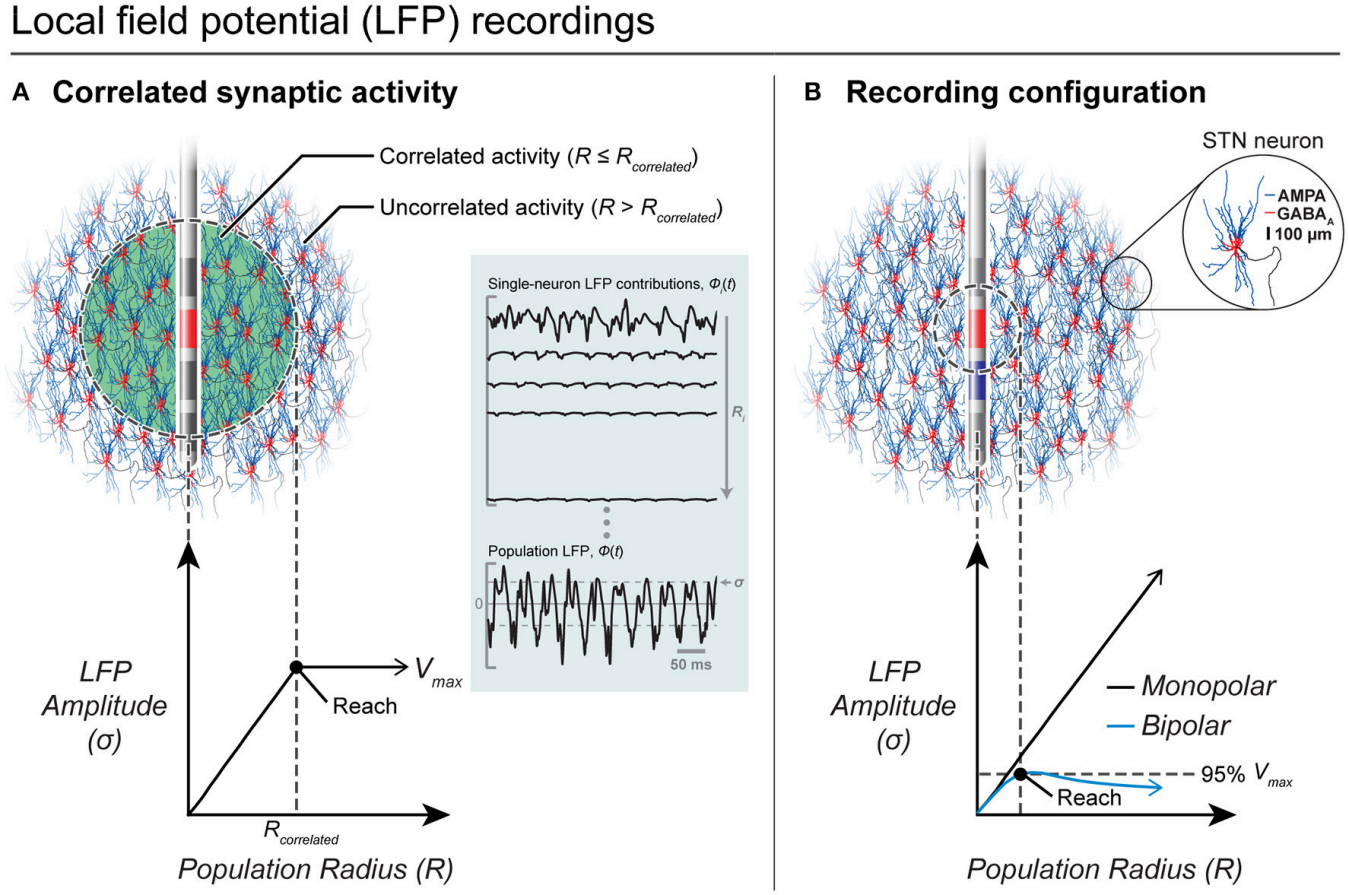
LFP recordings from DBS electrodes. **(A)** Correlated synaptic activity dominates the LFP and determines its spatial reach. A DBS electrode is shown implanted in the center of a volume of STN neurons. Neurons within a population radius (*R* ≤ R_correlated_) receive highly synchronous synaptic inputs while the remaining neurons (*R* > R_correlated_) receive uncorrelated synaptic inputs. Within correlated regions, an increase in the population radius produces a linear increase in the amplitude of the LFP. Outside of the correlated volume, there is no significant increase in the LFP amplitude. **(B)** Recording configuration effects on the LFP. In this example, all neurons receive correlated synaptic inputs. For a monopolar recording (red electrode only), the LFP amplitude increases linearly with an increase in the population radius and does not converge to a maximum value. However, a bipolar recording (red electrode—blue electrode) limits the amplitude and spatial reach of the LFP recording.

In general, the LFP is believed to be dominated by postsynaptic currents (Kajikawa and Schroeder, 2011). Previous experimental studies with intracortical microelectrodes suggested that the LFP only extends a few 100 μm (Katzner et al., 2009) while contradictory evidence suggest that the LFP can extend several millimeters (Kajikawa and Schroeder, 2011). A computational modeling study showed that the LFP spatial reach is not simple or stationary, but depends on a number of variables, such as neuron morphology, and the distribution and correlations in synaptic activity (Linden et al., 2011). A computational model to specifically characterize LFP recordings from DBS electrodes was previously developed (Lempka and Mcintyre, 2013). Using this model, it was determined that the LFP can extend several millimeters and that its spatial reach was dependent on factors such as the spatial distribution of correlated synaptic activity and the recording configuration, but was independent of the electrode impedance (see Figure 7).

To further increase the clinical utility of this modeling approach, a patient-specific LFP model to estimate the region of beta hyper-synchrony within the STN was developed. The model demonstrated that the size and shape of correlated activity within the STN and its relative location to the DBS lead dramatically affect the recorded LFP. It is believed that this patient-specific modeling approach represents an excellent tool to study the neural underpinnings of clinical LFP recordings and to help provide the knowledge necessary to develop effective closed-loop DBS technologies.

### Highlights and future directions

- The origin of the LFP is poorly understood with conflicting information regarding LFP's spatial reach.
- A previous computational model determined that the LFP can extend several millimeters and that its spatial reach was dependent on factors, such as the spatial distribution of correlated synaptic activity and the recording configuration, but was independent of the electrode impedance.
- A new computational model demonstrated that the size and shape of correlated activity within the STN and its relative location to the DBS lead dramatically affect the recorded LFP.
- Understanding the neural mechanisms of clinical LFP recordings is necessary to develop effective closed-loop DBS technologies. Future research will focus on determining the LFP construct, LFP interpretation based on electrode position, changes in new directional leads and variation over time.

### Coupled theoretical and experimental analysis of DBS electrodes

**Figure 8:**
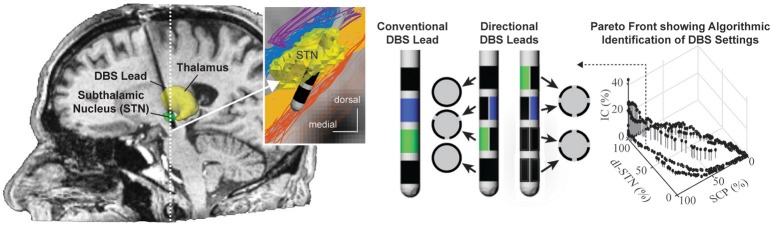
Programming of directional DBS lead designs. Conventional and directional DBS lead designs targeting the subthalamic nucleus for the treatment of Parkinson's disease. Computational programming algorithms can accommodate multiple regions of interest such as the dorsolateral subthalamic nucleus (dl-STN) and the superior cerebellar peduncle (SCP) as well as multiple regions of avoidance, i.e., the corticospinal tract within the internal capsule (IC). Shown is an example of a Pareto Front resulting from the particle swarm optimization algorithm approach to identify stimulation settings that maximize activation in regions of interest and minimize activation of regions of avoidance. Images courtesy of Edgar Peña, Julia Slopsema, and Matthew Johnson (with permission).

Nearly all DBS leads implanted to date consist of a stack of four cylindrical electrode contacts. More recently, directional DBS leads with electrode contacts segmented along and around the lead body have shown utility for more selective targeting of neural pathways of interest while avoiding activation of neural pathways implicated in DBS side-effects (Buhlmann et al., 2011; Martens et al., 2011; Keane et al., 2012; Zitella et al., 2013; Pollo et al., 2014) (See Figure 8). Computational models that integrate realistic tissue bioelectrics and cellular biophysics provide a useful framework to assess the design and performance of these directional DBS lead designs.

While many directional DBS lead designs have been proposed, computational models suggest that designs incorporating 3–4 radial electrodes used in concert with multi-channel independent current-controlled stimulation provide a good balance of (1) maintaining safe current density limits when using smaller electrodes at therapeutic stimulation amplitudes; and (2) requirements for higher channel counts to enable better shifting and sculpting of the activation volumes around a DBS lead (Teplitzky et al., 2016; Figure 8). Further, computational models have also shown that directional DBS leads can move the center of mass of axonal activation by (at most) 1–1.3 mm tangential to the lead shaft (Teplitzky et al., 2016). This suggests that directional DBS leads, while an enabling technology, may not be able to rescue therapy for DBS leads that are implanted significantly (>1–1.3 mm) off target. Models also suggest that axonal tracts oriented parallel, in comparison to perpendicular, to the applied electrical field will have lower activation thresholds (Lehto et al., 2017). Such orientation selectivity might help with structural leads design and avoid adverse side effects with DBS.

Once a DBS lead is implanted, stimulation settings are typically tested in a trial-and-error process by stimulating through combinations of electrode contacts to identify stimulation settings that optimize the therapeutic effect based upon clinical outcome measures (Volkmann et al., 2006). Recent developments in subject-specific computational bioelectric models and visualization of the predicted neuronal pathways activated have shown promise in identifying DBS settings that avoid inducing side effects (Frankemolle et al., 2010; Chaturvedi et al., 2013) Leveraging these computational models, several novel semi-automated programming algorithms have been advanced to assist with the selection of stimulation parameters. These algorithms have a basis in machine learning (Chaturvedi et al., 2013; Teplitzky et al., 2016), convex optimization theory (Xiao et al., 2016), and most recently particle swarm optimization (Peña et al., 2017), which allow clinicians to use multiple objectives in their identification of optimal DBS settings.

### Highlights and future directions

- Computational models support the safety of designs incorporating 3–4 radial electrodes used in concert with multi-channel independent current controlled stimulation.
- Computational bioelectric models and visualization of the predicted neuronal pathways activated have shown promise in identifying DBS settings to deliver therapy and avoid inducing side effects.
- Orientation selective DBS that leverages directional lead technology is poised to improve clinical outcomes (Lehto et al., 2017).
- Future clinical implementation of direction leads will likely leverage novel semi-automated programming algorithms to assist with the selection of stimulation parameters.

## DBS targeting and metrics

### Noninvasive biomarkers to advanced emerging DBS electrode technologies

Directional DBS electrode technology, as available from multiple vendors, is now entering the commercial market. However, there is a lack of robust tools with which to efficiently implement increasingly adaptable and complex DBS systems. To address this problem, new putative biomarkers to measure patient-specific cortical activation patterns elicited by DBS with combined electroencephalography and electrocorticography (EEG/ECoG) have been investigated.

Clinical applicability of effective contacts with a new directional lead incurs a number of challenges, including the number of contacts to be used, potential combinations of contacts, and complex interactions with anatomical structures. Therefore, a goal is to innovate new approaches to tailor DBS programming adjustments in individuals and to more rapidly arrive at effective, well-tolerated stimulator settings with directional lead technology by using software to remove stimulus artifacts. This should facilitate measurement of fast dynamics of brain responses where there is a short latency to stimulation of the cortex. Such knowledge will be important and useful (1) to better understand the concept of DBS dose, and this may have broad applications for the development of minimally invasive biomarkers; (2) to refine surgical targeting of the DBS electrode in real time; and (3) to inform emerging closed loop stimulation strategies.

### Highlights and future directions

- Technological advances are aimed at developing adaptable devices and biomarkers to measure patient-specific cortical activation patterns elicited by DBS with combined electroencephalography and electrocorticography (EEG/ECoG).
- There is a need to continuously develop and improve effective and simple stimulation settings, surgical targeting, and manipulation of the field of stimulation.
- Questions remain regarding the effects of different stimulation settings among different structures in the basal ganglia (and elsewhere).

### Temporal pattern of stimulation is a new dimension of therapeutic innovation

In the course of experiments intended to test the hypothesis that the reductions of symptoms by DBS required regularization of the firing patterns of neurons (Grill et al., 2004), it was discovered that the effects of DBS were strongly dependent on the temporal pattern of stimulation. Specifically, random patterns of subthalamic nucleus DBS were not as effective as regular frequency DBS at relieving motor symptoms in the 6-OHDA lesioned rat model of PD (Mcconnell et al., 2016) or in humans with PD (Dorval et al., 2010). Similarly, random patterns of thalamic DBS were not as effective as regular frequency DBS at relieving tremor in persons with essential tremor (Birdno et al., 2007, 2008, 2012). In addition to supporting the importance of regularization of neural firing to the efficacy of DBS, this finding inspired the idea of explicitly designing temporal patterns of stimulation to increase the efficacy and energy efficiency of DBS.

However, the design space for temporal patterns is enormous—for example a 200 ms duration train, composed of 1 ms bins each which may or may not contain a pulse, results in 10^50^ different possible patterns of stimulation. Therefore, a model-based design approach employing computational evolution to design optimized temporal patterns of stimulation was developed. Computational evolution works analogously to biological evolution, and the organisms in this approach are temporal patterns of stimulation, and the fitness of any particular pattern is evaluated using a model-based proxy for symptoms (Brocker et al., 2017). Thus, an innovative approach to make a temporary direct connection to the brain lead during surgical replacement of the battery-depleted implantable pulse generator (IPG) (Swan et al., 2014), and to conduct short-term intraoperative testing of the model-optimized patterns. The results demonstrated that the optimized patterns either addressed bradykinesia more effectively than conventional regularly patterned DBS (Brocker et al., 2013), or enabled equivalent treatment of bradykinesia but with a substantial reduction in the required energy (Brocker et al., 2017). This latter finding is important, as it enables increases in the battery life of IPGs, reduction in IPG size, or longer intervals between recharging.

Subsequently a start-up company, Deep Brain Innovations, Inc. conducted a multi-center double-blinded trial comparing temporally optimized patterns of stimulation to conventional high frequency DBS. The results of this study demonstrated that temporally optimized patterns of stimulation produced equivalent or better symptom reduction and substantially reduced energy requirements. In addition to their promise to improve DBS therapy for PD, these data suggest that the temporal pattern of stimulation might be a new dimension of therapy parameter adjustment that can be exploited in other applications of neuromodulation.

### Highlights and future directions

- The effects of DBS were strongly dependent on the temporal pattern of stimulation and computational models can provide optimized patterns to improve efficacy or improve efficiency.
- Optimized patterns improved bradykinesia more effectively than conventional regularly patterned intraoperative DBS.
- Preliminary results showed that stimulation was well tolerated and produced equivalent or better symptom reduction and substantial reduced energy requirement in some patients.

### Using human connectome data to map deep brain stimulation targets

Stereotactic targeting of the anterior limb of the internal capsule (ALIC) has been used for decades to treat patients with refractory OCD, depression, and other neuropsychiatric disorders. However, there is uncertainty about optimal targeting within the ALIC, as different locations appear to have variable efficacy/effectiveness. Using diffusion tensor imaging (DTI), the ALIC was anatomically segmented based on prefrontal connectivity in order to evaluate the effect of various stereotactic targets.

**Figure 9:**
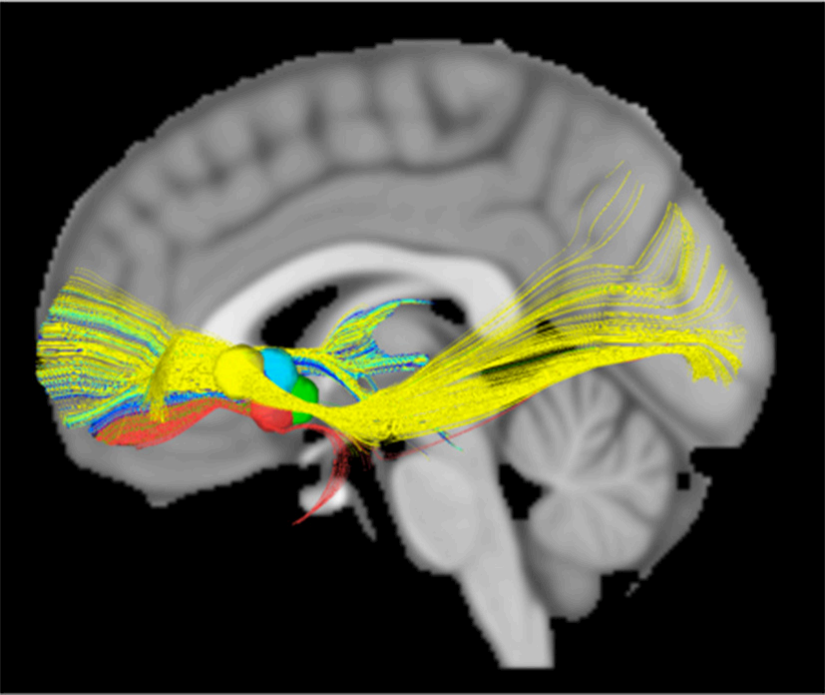
DTI modeled structural connectivity. Tracts running through the five modeled targets (colored spheres in the diagram) according to the HCP 842-subject diffusion data template.

ALIC segmentations based on frontal Brodmann area (BA) connectivity were generated and combined for 40 subjects from the Human Connectome Project (HCP) using connectivity-based seed classification (Nanda et al., 2017). A literature review revealed five stereotactic targets within the ALIC. Targets were modeled as 5 mm spheres and were evaluated for overlap with various DTI-defined ALIC segments. Deterministic tractography was performed on an 842-subject HCP DTI template using modeled targets as seeds to identify involved connectomic networks (Figure 9).

All 40 ALIC segmentations exhibited a dorsal-ventral axis of organization. On average, the combined segmentation was accurate for 66.2% of individuals. The region assigned to BA11 (orbitofrontal cortex, OFC) exhibited the greatest consistency across individuals, with 12.1% being consistently assigned in all 40 subjects. According to the segmentation, a mean of 63.9% of modeled lesion volume within the ALIC intersected with the BA11 region. All five modeled targets exhibited connectivity to OFC in the 842-subject HCP template.

These results clarify the organization and variability of the ALIC. This variability suggests that patients may benefit from pre-operative tractography for individualized targeting, although current stereotactic targets tend to involve the most consistent ALIC sub-regions. These findings also suggest that stereotactic targeting within the ALIC likely involves modulation of prefrontal-subcortical tracts connecting the OFC, which bears relevance to the cortico-striato-thalamo-cortical (CSTC) model of neuropsychiatric pathophysiology.

### Highlights and future directions

- The Anterior limb of the internal capsule (ALIC) is a frequent DBS target for treatment of psychiatric diseases.
- Using a DTI-based template from the Human Connectome, a study was undertaken to map this region to better understand these tracts for DBS application.
- This research demonstrated that the ALIC has a general axis of orientation but variability is present and can be probabilistically quantified (some loci show differences).

### RAD-PD: registry for the advancement of DBS in Parkinson's disease

PD DBS is well known to improve motor function and quality of life in patients experiencing motor complications. However, numerous questions remain about best practices related to DBS, which cannot be answered through traditional clinical trials methods. The Registry for the Advancement of DBS in Parkinson's Disease (RAD-PD) has been proposed as a partnership between the Parkinson Study Group (PSG), Neuropoint Alliance (NPA) and Neurotargeting, LLC, to establish a quality improvement (QI) registry for DBS.

**Figure 10:**
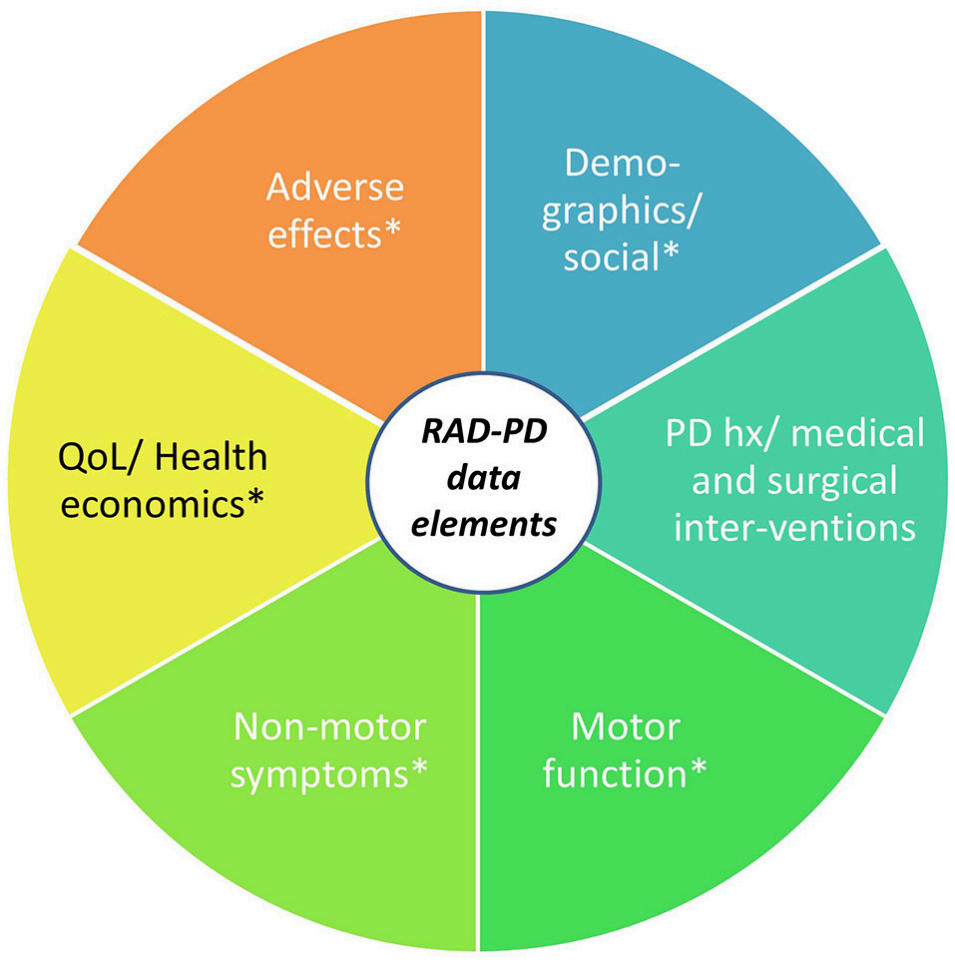
RAD-PD: Registry for the Advancement of DBS in Parkinson's Disease. The RAD-PD data elements include clinician-measured and patient-reported outcomes (indicated by ^*^), which will be collected at baseline (pre-operatively), 6 months, 1 year, and annually through 5 years. The total project period will last 10 years.

The QI design of RAD-PD will allow for continuous benchmarking of selected data and the opportunity to review those findings via regular dash-boarding and discussion at periodic study group meetings. The registry can support research functions, secondary analysis, linkage to other databases, sub-studies analysis, data collection and use of a de-identified dataset to answer research questions. Multiple clinical questions that cannot otherwise be practically answered will be investigated regarding the best practices surrounding DBS therapy, adverse effects of DBS (and their determinants), the health economics of PD DBS, as well as the correlates of disparities in outcomes among individuals receiving the therapy (Figure 10).

The project design has potential to gather longitudinal, prospective data from over 1,000 participants with PD across a 5-year period of DBS therapy, with a data collection and analysis period of up to 10 years. The project is currently under review for funding. Once implemented, the potential exists to extend the RAD-PD infrastructure for the capture and analysis of similar data from patients with other conditions being treated with DBS.

### Highlights and future directions

- The Registry for the Advancement of DBS in Parkinson's Disease (RAD-PD) is a DBS Registry for PD to address numerous clinical questions.
- The registry would integrate different groups for analysis including Neurotargeting, Neuropoint Alliance, and the Parkinson's Study Group (PSG).
- The registry will focus on a Quality Improvement Registry utilizing a data-driven approach.

### Diagnostic data management in closed-loop cortical stimulation for epilepsy

**Figure 11:**
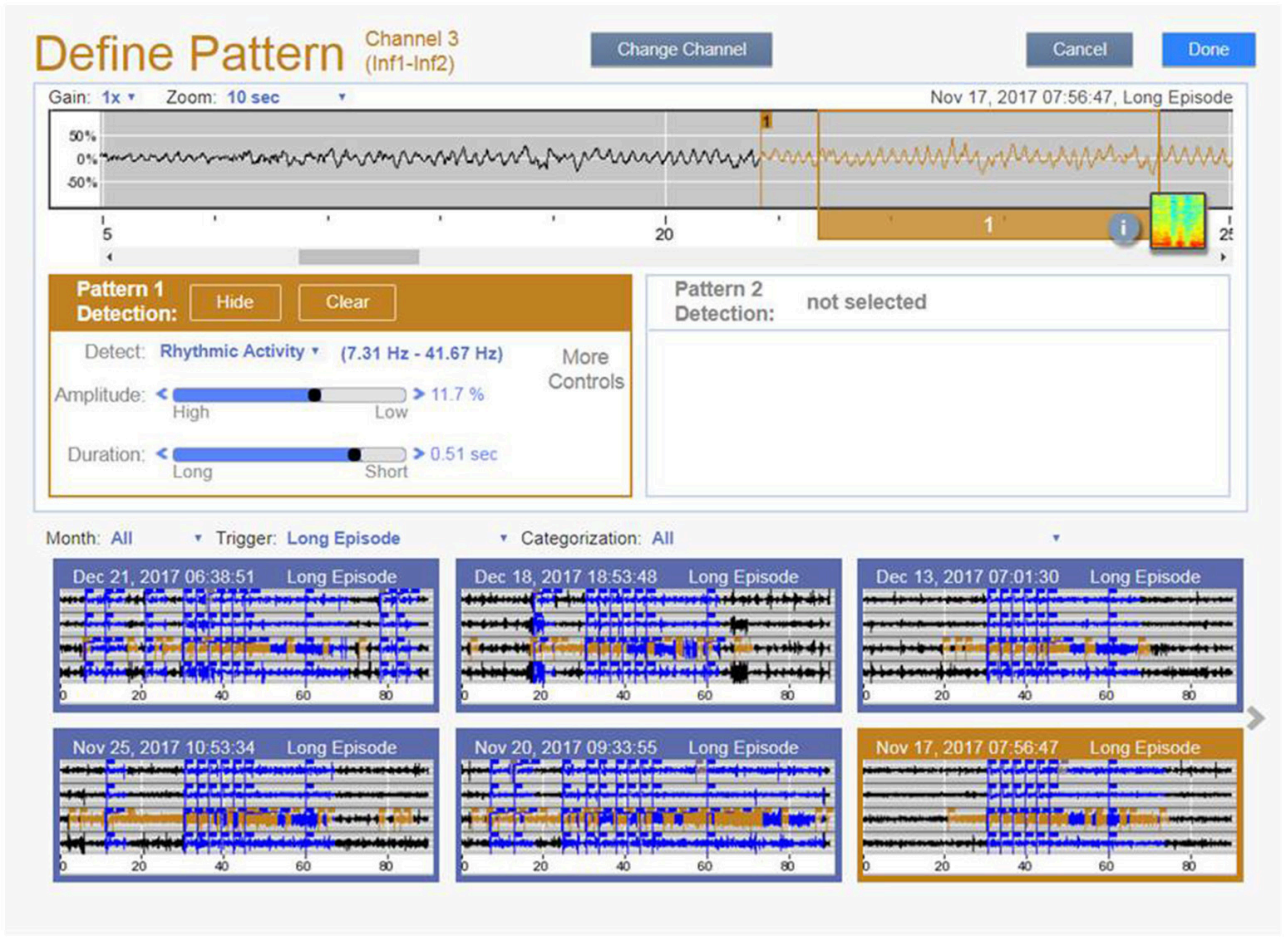
Defining Pattern feature in PDMS. The clinician clicks on the ECOG in the top panel and the PDMS suggests initial detection settings that are immediately simulated in the bottom panel. Sliders in the middle panel allow the physician to make adjustments to achieve the desired performance.

The RNS® System is an FDA approved therapy for treating medically refractory partial onset epilepsy in individuals 18 years of age or older (Bergey et al., 2015). The RNS System consists of an implantable neurostimulator and leads (depths or strips), a physician operated programmer, a patient operated remote monitor and a secure website referred to as the Patient Data Management System (PDMS). The neurostimulator records data of frequency, timing, and location of electrographic activity specific to each patient. The neurostimulator also stores electrocorticograms (ECOGs) as well as counts and timing of various events (detected patterns, long duration events, high amplitude events and magnet placements). These data are collected via the physician programmer and the patient operated remote monitor to provide a long-term record of patient electrographic activity. These data are then organized and made available for clinician review via the PDMS (Figure 11).

In the clinical care environment, physicians must be able to quickly assess data trends and make programming changes within the constraints of a typical clinic visit. The PDMS provides concise summary data and capabilities to quickly sort and view classes of ECOGs and graph trends of various events. It also allows physicians to determine detection settings by clicking on ECOG locations of epileptiform activity they intend to detect. The system then runs a series of algorithms to suggest initial settings. The clinician can rapidly review the suggested settings performance against the library of stored ECOGs and make adjustments to achieve the detection performance desired.

### Highlights and future directions

- NeuroPace PDMS (Patient Data Management System) allows remote storage on data bank and can simulate detection rate based on the parameter selection and previously data.
- The PDMS provides concise summary data capabilities to quickly sort and view classes of ECOGs and to graph trends of various events to determine detection settings by clicking on ECOG locations of epileptiform activity.

## Federal initiatives

### Updates on DBS directions from the NIH P50 Udall program

NIH Udall Center grants aim to foster multidisciplinary work with nine centers around the United States. Despite years of clinical success and efforts to improve clinical outcomes, the degree of therapy achieved with DBS varies widely among patients for both STN DBS and GPi DBS (Deep-Brain Stimulation for Parkinson's Disease Study Group et al., 2001). Such variability across centers and within any given center likely stems from multiple factors, including patient phenotype(s), assessment protocol, patient health, DBS target, DBS lead location within the target, and the stimulation parameters used to deliver the therapy (Kleiner-Fisman et al., 2006).

To address these challenges, the NIH recently awarded a Morris K. Udall Center of Excellence for Parkinson's Disease Research grant to the University of Minnesota (PI: Jerrold L. Vitek). The University of Minnesota's (UMN) NIH Udall Center is focused on understanding the electrophysiological features that underlie individual motor signs of PD and developing new DBS strategies to treat these motor signs more effectively and more consistently. This is being accomplished in humans through a combination of intra-operative microelectrode, externalized lead recordings, post-operative LFP recordings using the Medtronic RC+S “Brain Radio,” and DBS therapy implemented through the lens of novel targets and stimulation paradigms. These data will be complemented by electrophysiological studies in preclinical animal models of Parkinson's disease with directional DBS implants, and by the development and electrophysiological characterization of optimization tools for improving subject-specific precision of DBS therapy.

### Highlights and future directions

- The UMN NIH Udall Center is focused on understanding the electrophysiological features that underlie individual motor signs of PD and in developing and advancing new DBS targets and stimulation paradigms through a combination of intra-operative microelectrode and post-operative LFP recordings.
- Specific goals of the UMN Udall Center include:
  ◦ Project 1 (Human): Understand PD pathophysiology as it relates to DBS.
  ◦ Project 2 (Human): Identify mechanisms and pathways mediating the motor effects of pallidal DBS.
  ◦ Project 3 (Pre-clinical Research): Identify electrophysiological mechanisms underlying the clinical variability with DBS therapy for Parkinson's disease.

### High bandwidth wireless interfaces in quadraplegia

The BrainGate consortium is a multi-center effort to restore communication and movement capabilities to people with paralysis and to develop next generation neurotechnologies. The study has recruited 13 participants with over 9,310 total implant days. A completely implantable brain computer interface has also been developed for potential future human use, toward neurally controlled point-and-click (keyboard, communication devices) and for multi-dimensional control of robotic assistive devices or one's own arm and hand. Patients with severe neurological motor disability from ALS, brainstem stroke, or cervical spinal cord injury have demonstrated impressive control of communication and mobility technologies. The core technology, consisting of the decoding of ensembles of single neurons, could provide a neurophysiological signature that could be deployed as part of a closed loop neuromodulation device in both neurologic or psychiatric disorders. Wireless arrays record broad band intracortical physiology and are able to provide external responsive stimulation to contracting implanted muscles. In the future, wirelessly connected cortical arrays capable of recording broadband intracortical signals will also be able to direct the movement of functionally electrical stimulation systems for the restoration of limb movement. The long term aims of the BrainGate trial are to neuroengineer improved BCI capabilities; communicate the validity and viability of BCI-based approaches to both the medical community and representative stake-holders (i.e., patients and the public), and assist and restore function to patients in need.

### Highlights and future directions

- BrainGate is a multicenter effort dedicated to restoring movement capabilities to motor-impaired individuals. The initiative initially focused on achieving decoding accuracy, and this is being extended by research into improvements in the efficiency of filter calibration and adaptation.
- Future challenges include designing of devices that are portable, fully implanted, compact, wireless, and available around-the-clock to support useful applications and activities of daily living.
- Important questions and challenges remain regarding the number and configuration of electrodes, methods to assess the stability of decoders, personal assessments (to each patient) that balance the risks vs. potential performance as compared the risk/benefit provided by BCIs that record from the scalp.

### Updates from DARPA: restoring active memory (RAM)

#### Neurotechnology: bridging the gap between mind and machine

DARPA's innovations in neurotechnology are making possible real-time, seamless translation between human brains and machines. These technologies have enabled initial approaches that enable analyses of the ways that diffuse and varied signals from arrays of firing neurons affect brain function. DARPA's current investments in neural interfaces and related technologies build upon this understanding of neural encoding and decoding, developing multi-scale computational models with high spatial and temporal resolution to target a variety of neurologic functions, and these research approaches are synergized by studies that address engineering challenges of designing implantable, closed-loop systems. Key DARPA-funded capability demonstrations include controlling and receiving feedback from a prosthetic arm using sensors in the motor and somatosensory cortices; receiving feedback from a virtual hand using implants in peripheral nerves; and improving declarative memory through stimulation of specific brain regions. DARPA's work is establishing a basis of what is currently possible, revealing how neurotechnology could facilitate symbiotic human-machine interfacing, and engaging assessment and address of the ethical, legal, social, and policy issues fostered by emerging neurotechnologies (see also, below).

#### Modulating human memory using direct brain stimulation

The Restoring Active Memory (RAM) project aims to develop implantable therapies to treat veterans with TBI by increasing their ability to encode information. In this study, the hypothesis that targeted electrical stimulation can modulate neural encoding states and subsequent memory outcomes was tested. Using recordings from neurosurgical epilepsy patients with intracranially implanted electrodes, multivariate classifiers were trained to discriminate spectral activity during the encoding of words that predicted whether patients would later remember or forget these words; and stimulation was applied to various brain regions to modulate performance.

**Figure 12:**
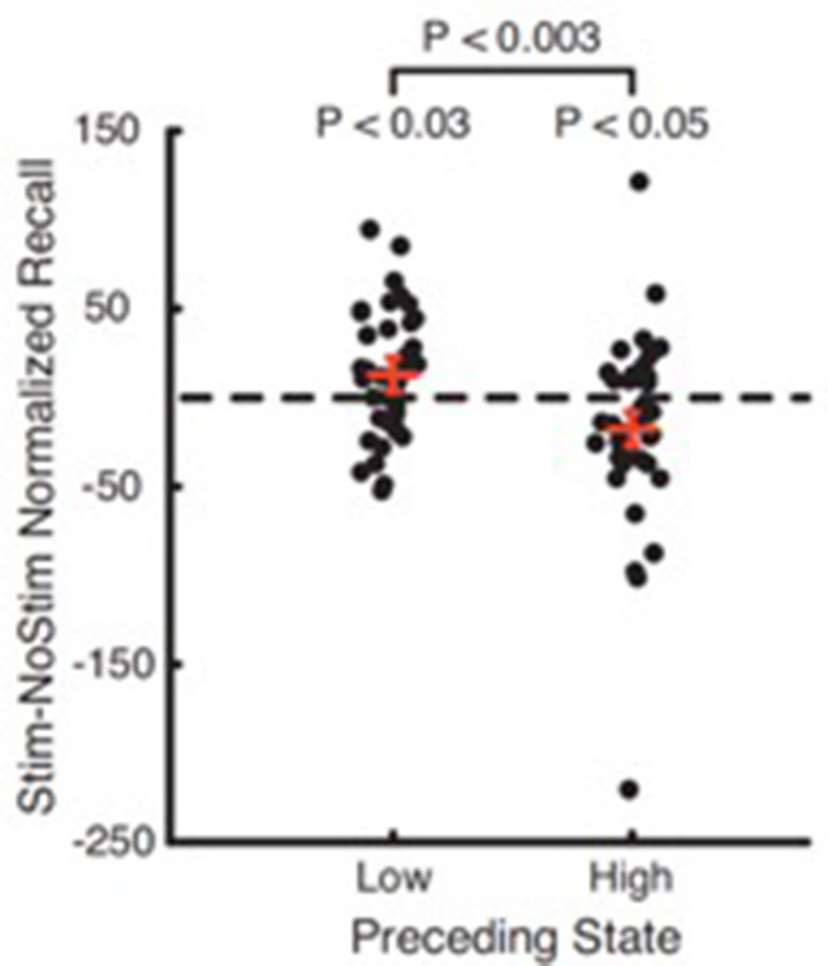
The effect of stimulation is dependent on brain state. Recall performance increased if stimulation was delivered when the brain was in a low encoding state (*p* < 0.03) and decreased if delivered in a high encoding state (*p* < 0.05). The difference between low and high stimulation was also significant (*p* < 0.003). Red bars show mean SE of the difference.

It was found that stimulation modulates performance, with large variability across the population and across the various brain regions stimulated. Hippocampal stimulation tended to impair recall performance while lateral temporal cortex stimulation significantly improved recall performance. In addition, stimulation increased recall performance if delivered when the classifier indicated low encoding efficiency but had the reverse effect if stimulation was delivered when the classifier indicated high encoding efficiency (Figure 12). These data suggest strategies for therapeutically treating memory dysfunction using closed-loop brain stimulation.

### Highlights and future directions

- Electrical stimulation can modulate neural encoding states and subsequent memory outcomes.
- The timing of stimulation and strict anatomical targeting appear critical to enhance memory. Developing patient-specific classifiers and stimulation patterns that adjust over time might provide sustained benefits.
- Current work provides proof of concept for the future of cognitive enhancement in other areas, including attention or focus, using closed-loop neurostimulation technologies.
- Future steps include determining the number of contacts needed for sensing and stimulation, and the translation of these technologies into patients with mild cognitive impairment (MCI) and/or Alzheimer's dementia (AD).

#### Hippocampal memory prostheses: neural code-based DBS

A hippocampal memory prosthesis is defined as a closed-loop system that bypasses damaged hippocampal region(s) to restore or enhance memory functions (Berger et al., 2011). Like closed-loop DBS systems, it consists of a recording unit (e.g., multi-electrode array), a signal processing/control unit, and a stimulator (e.g., stimulating electrodes). Differing from DBS systems, which typically deliver stereotypical stimulation patterns (e.g., HFS or LFS) to target regions to modulate neural activities, hippocampal memory prostheses utilize neural code-based stimulation patterns to reinstate neural signal transmission and thus mimic brain functions. Hippocampal memory prostheses have been developed and tested in rodents (Song et al., 2007, 2009; Berger et al., 2011, 2012; Hampson et al., 2012a,b), nonhuman primates (Hampson et al., 2013), and human epilepsy patients (Song et al., 2016; Hampson et al., in preparation).

**Figure 13:**
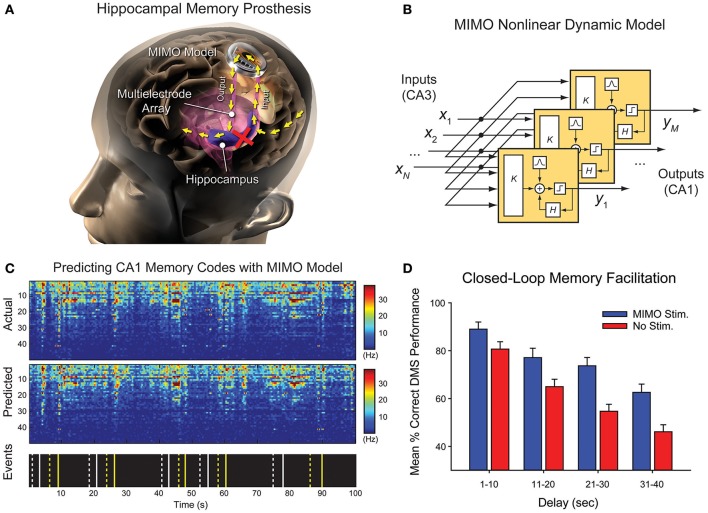
Hippocampal memory prosthesis. **(A)** Hippocampal prosthesis restores memory functions by reinstating neural signals and thus bypassing damaged brain region. **(B)** A MIMO model serves as the computational basis of hippocampal memory prostheses. **(C)** A MIMO model accurately predicts CA1 (output) codes based on CA3 (input) codes in human. **(D)** MIMO-stimulation enhances memory functions in preclinical animal testing.

This technique is now being applied to human studies in which multi-electrode “macro-micro” depth electrodes are implanted in the hippocampus of epilepsy patients undergoing Phase II invasive monitoring for seizure localization (Figure 13). Hippocampal CA3 and CA1 neural ensembles are recorded while patients perform a delayed match-to-sample (DMS) task. Multi-input, multi-output (MIMO) nonlinear dynamical models are built to describe the transformation from CA3 (input) spatio-temporal patterns (codes) of spikes to CA1 (output) spatio-temporal patterns (codes) of spikes using CA3 and CA1 data recorded from success trials of DMS tasks, and further drive stimulations to the CA1 region (Song et al., 2013, 2016). In combination with the modeling from human data, results from preclinical testing in rodents and nonhuman primates (Hampson et al., 2013) demonstrate that (1) MIMO models accurately predict CA1 codes in real-time from ongoing CA3 codes; and (2) closed-loop electrical microstimulation of CA1 using the MIMO-predicted CA1 codes improves DMS performance in those same subjects, indicating improvement of working memory function.

### Highlights and future challenges

- Hippocampal neurostimulators implanted in epilepsy patients, multi-input, multi-output (MIMO) nonlinear dynamical models have been built to describe the transformation from CA3 (input) spatio-temporal patterns (codes) of spikes to CA1 (output).
- MIMO models accurately predict CA1 codes in real-time from ongoing CA3 codes and improve DMS performance and delayed recognition of DMS visual stimuli.
- An increased level of complexity in memory decoding is expected going forward. Certain limitations apply to current stimulating electrode and improved electrode design might be necessary to facilitate research.
- Future research to determine the most effective location for stimulation, changes in plasticity and stimulating parameters is needed.

## Ethical and policy issues

As evidenced in the this report, there are ample new developments in DBS technology. These include increasingly sophisticated electrodes and electrode arrays (that enable both stimulation and recording), iterative BCI systems' hard- and software, and closed-loop adaptive systems. As well, a building body of research—as presented here - demonstrates the efficacy of various forms of DBS in mitigating signs and symptoms of disorders beyond PD, dystonia and epilepsy (e.g., Tourette syndrome; depression; OCD; memory loss in TBI and AD). This suggests, if not supports, an expanding translational viability and potential value of such approaches. This widening scope of capability and use fosters ethical and policy issues, which can be parsed into (1) those inherent to the characteristics of the technology and/or technique, and (2) those derived from the uses of such technologies in medicine and other applications in the social sphere (for complete address of specific neuroethico-legal and social issues, see: Buniak et al., 2014; Darragh et al., 2015; Giordano, 2017). These are not mutually exclusive: the relative novelty of technologies and techniques spawns questions about the intermediate and ongoing safety and effects in practical use (Giordano, 2015). The prompts questions of if and when these approaches will represent an accepted standard of care for certain disorders; where and under what conditions/contingencies DBS will be situated in the plan/algorithm of care for these pathologies, and if and to what extent medical and/or socio-economic and legal means of support will be provided to enable such care—in both the short and long term. Current problems in the subsidy of DBS treatment of certain movement and psychiatric disorders undergird the importance and need for continuing discourse and deliberation focal to these issues (Rossi et al., 2017).

As well, questions persist as to whether and how implantable neuromodulation might affect aspects of neuropsychological function that are associated with identity, “free will” and autonomy, and what this incurs and infers for the ethically sound use of DBS both to treat defined medical conditions, as well as to potentially optimize/enhance particular aspects of cognition, emotion and/or behavior (Giordano, 2015). We have posited that any new developments in neurotechnology entail effort to define and address the neuroethico-legal and social issues that may, and are likely to be generated by such research and its trsanslation in medical and/or other applications (Shook and Giordano, 2015), and unapologetically reiterate that assertion here. To effect such effort, it will be therefore important—and necessary—to both employ extant ethical and policy constructs and to revisit, and in some cases revise these concepts and processes so as to better meet the exigencies borne of emerging technology and techniques, and of the social contingencies and concerns that affect and are affected by their use in international contexts (Shook and Giordano, 2014; Giordano, 2015). Our ongoing work remains dedicated to these tasks.

## Summary and conclusion

**Figure 14:**
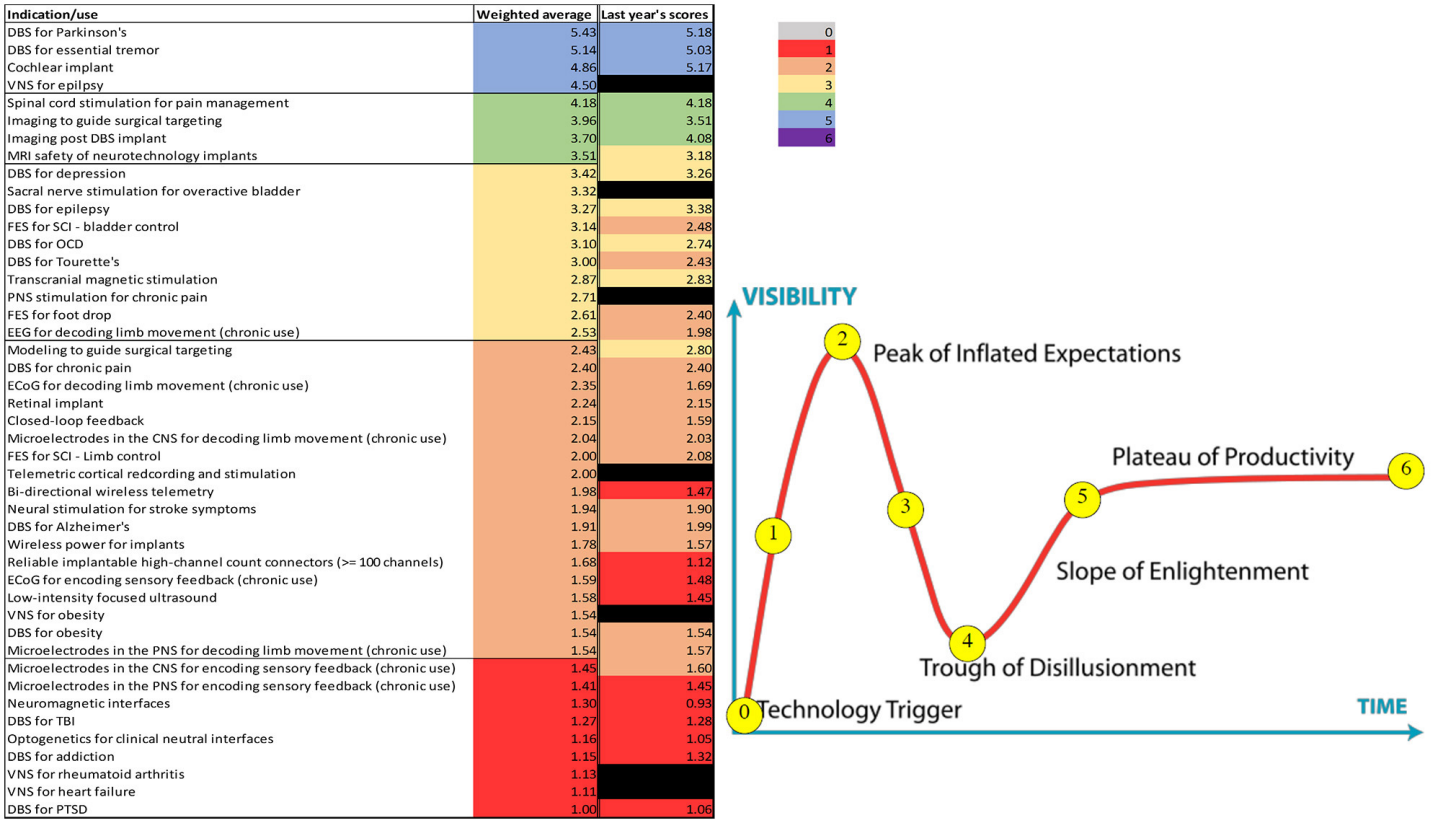
Representation of the anonymous annual survey results, polling the participants of the DBS Think Tank on the hype cycle positions of different DBS related neurotechnological advances and indications. On the right side, the hype cycle graph (adapted Jackie Fenn, “When to leap on the hype cycle,” Decision Framework DF-08-6751, Research Note, GartnerGroup RAS Services, June 30, 1999 with permission) that represents the different stages of development. On the left side, a table summarizing the weighted averages of the position of the different neurotechnological indications or uses on the hype cycle graph. Positions 0 to 6 are color coded as noted.

In this paper, we have provided views to the relevant topics and updates discussed at The Fifth Annual DBS Think Tank in Atlanta, GA. Similar to prior years' ThinkTank meetings, an anonymous 40 question poll was sent online to assess participants' perspectives and attitudes toward the current and near-term future developments and applications in the field. Sixty two participants responded. Figure 14 presents a summary of these responses, compares them to last year's responses, and depicts this year's responses as positioned in various points upon the hype cycle graph. It is notable that some participants' views of DBS applications (e.g., closed loop feedback) moved to the peak of inflated expectations, while, others (e.g., DBS for Tourette's) dropped from this position on the hype cycle graph, and yet others (e.g., Imaging to guide surgical targeting and spinal cord stimulation for pain management) remained in the trough of disillusionment. Consistently, the use of DBS for PD and essential tremor has reached the plateau of productivity and among most participants, cautious optimism remains regarding the use of neuromodulation for several neuropsychiatric conditions, non-neurological indications and the development of newer technologies.

In conclusion, the Fifth Annual DBS Think Tank provided a nexus for discussion and vector for the exchange of ideas about the current and near-term state and direction of DBS research, ongoing technological, scientific and clinical challenges and opportunities, and ethical and policy concerns and possible resolutions important to shaping the future of DBS research and use in practice.

## Ethics statement

Individual studies were approved by the local Institutional Review Board of participating institutions in this technical report and written informed consent was obtained from all participants.

## Author contributions

AR-Z, JG, AG, PB, JS, KF, LA, PhS, HB-S, WH, CM, WG, DK, WMG, HW, MJ, JV, DG, DR, DS, TB, RH, SD, LH, NS, PaS, GW, VT, HM, JJ-S, PN, SS, RG, SFL, LL, WD, and MO fulfilled the authorship criteria by substantial contributions to the conception of the work, providing data for the work, revisiting it critically for important intellectual content, approving the final version, and agreeing to be accountable for all aspects of the work in ensuring that questions related to the accuracy or integrity of any part of the work are appropriately investigated and resolved.

### Conflict of interest statement

The authors declare that the research was conducted in the absence of any commercial or financial relationships that could be construed as a potential conflict of interest.
